# Metrics for Assessing Cytoskeletal Orientational Correlations and Consistency

**DOI:** 10.1371/journal.pcbi.1004190

**Published:** 2015-04-07

**Authors:** Nancy K. Drew, Mackenzie A. Eagleson, Danny B. Baldo Jr., Kevin Kit Parker, Anna Grosberg

**Affiliations:** 1 Department of Biomedical Engineering, University of California, Irvine, Irvine, California, United States of America; 2 School of Engineering and Applied Sciences, Harvard University, Cambridge, Massachusetts, United States of America; 3 Department of Chemical Engineering, University of California, Irvine, Irvine, California, United States of America; UNITED STATES

## Abstract

In biology, organization at multiple scales potentiates biological function. Current advances in staining and imaging of biological tissues provide a wealth of data, but there are few metrics to quantitatively describe these findings. In particular there is a need for a metric that would characterize the correlation and consistency of orientation of different biological constructs within a tissue. We aimed to create such a metric and to demonstrate its use with images of cardiac tissues. The co-orientational order parameter (COOP) was based on the mathematical framework of a classical parameter, the orientational order parameter (OOP). Theorems were proven to illustrate the properties and boundaries of the COOP, which was then applied to both synthetic and experimental data. We showed the COOP to be useful for quantifying the correlation of orientation of constructs such as actin filaments and sarcomeric Z-lines. As expected, cardiac tissues showed perfect correlation between actin filaments and Z-lines. We also demonstrated the use of COOP to quantify the consistency of construct orientation within cells of the same shape. The COOP provides a quantitative tool to characterize tissues beyond co-localization or single construct orientation distribution. In the future, this new parameter could be used to represent the quantitative changes during maturation of cardiac tissue, pathological malformation, and other processes.

## Author Summary

Biological tissues are highly organized on multiple length scales. In tissue engineering, recreating the *in vivo* architecture is an important aspect of *in vitro* experiments. An ability to quantify organization of cellular constructs, specifically the correlation in their orientations, would greatly enhance both our understanding of the function of each construct and provide a tool to evaluate engineered tissues. In this work, we have developed a parameter to evaluate the correlation and consistency of construct orientation. We have extensively characterized this co-orientational order parameter analytically, validated it using synthetic data, and demonstrated its use with experimental data of Z-lines and actin fibrils architectures in engineered cardiac tissues. As long as the orientation angles and location of constructs are known, the parameter can quantify both orientational correlation and consistency. Thus, the co-orientational order parameter has a wide scope of application both in biology and potentially outside of it.

## Introduction

The architecture and organization of the cytoskeleton components in cells, the cells in tissues, and cellular ensembles in organs affect function at each of these physiological scales [1–4]. The study of architecture is therefore key to understanding how the cellular microenvironment potentiates function, and may provide new insights in the study of physiological mechanisms. Furthermore, for proper function, different components of the cytoskeleton, cell, or tissue need to co-localize and orient properly with respect to each other [5, 6]. Quantifying the degree of orientation of cells and subcellular components, both relative to themselves and to other components, is thus crucial for evaluating the quality of engineered tissues [7].

The problem of describing the organization of biological structures is twofold: first, the orientation of the constructs needs to be quantified from the available images, and second, a metric needs to be applied to summarize the overall organization. The quantification of orientation from biological images is in principle straightforward and can be either done manually [8] or with a variety of computational algorithms [6, 9–11]. As far as the second problem is concerned, summarizing the overall organization after image analysis involves selecting a metric, which is more controversial. As a result, a wide variety of metrics are utilized in the bio-imaging field. For example, some assume that the parameter can be described as the standard deviation of a truncated Gaussian, or normal, distribution [12]. Others use the von Mises circular distribution [9, 10], which is a wrapped normal distribution. However, cellular and cytoskeleton distributions are often non-Gaussian, and their being non-Gaussian may be of crucial importance [13].

An alternative metric, the Orientational Order Parameter (OOP), has been developed in the field of liquid crystals. The OOP is a mathematical construct developed to quantify the degree of order in anisotropic medias [14]. Mathematically, the OOP is equivalent to resultant vector length from the circular distribution with a period of *π* [15]. In biology, the OOP has been successfully employed to characterize organization of bacteria [16], fibroblasts [17], vascular smooth muscle [18], actin fibrils alignment in valve endothelial cells [19], and Z-lines in cardiac muscle [20]. However, there is a lack of a robust correlation metric that has been characterized for use with biological images. The suite of correlation parameters provided by circular statistics are either too limited to be used with cytoskeleton organization or so complex the results are hard to connect back to biological phenotypes [21]. Other correlation metrics are also not ideal for correlating orientations of the cytoskeleton components, and to date no metric has been developed or specifically characterized for this purpose [22–24].

In this work, we develop a new parameter with similar mathematical framework as OOP that will characterize both consistency of orientation of a single component and correlation of orientation of two components. As an example, we apply this Co-Orientational Order Parameter (COOP) to compare orientation of Z-lines and actin filaments in a neonatal rat ventricular myocyte (NRVM) monolayer. Lastly, we show how the COOP can be used to measure the consistency of building a cardiomyocyte on a triangular island of extracellular matrix (ECM).

## Results

### Theoretical Results

**Fig 1:**
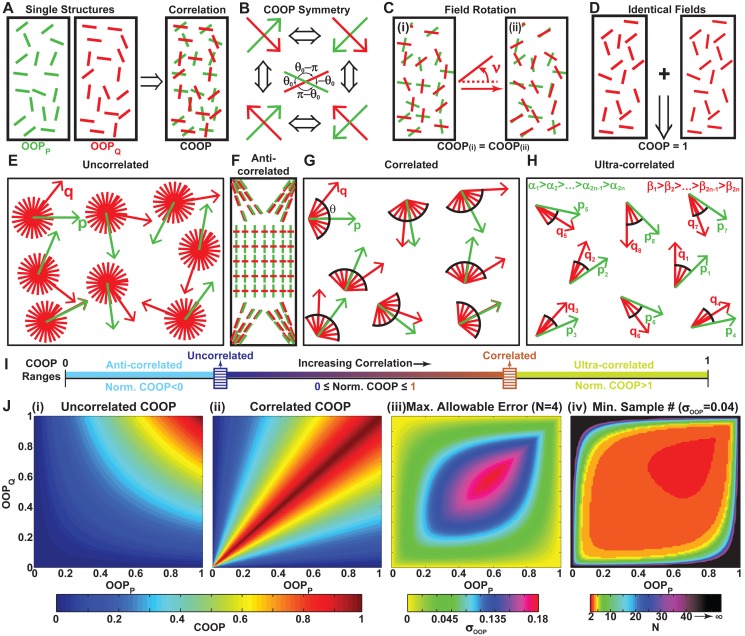
Theoretical formulation of the co-orientational order parameter (COOP). (A) The parameter is based on two independent sets of pseudo-vectors that are co-localized in space; (B) the COOP is symmetric to all permutations of 180° symmetry associated with the pseudo vectors; (C) the COOP remains the same if every pseudo-vector in one of the fields is rotated by the same angle; (D) if two identical fields of pseudo-vectors are compared to each other, *COOP* = 1; (E) The uncorrelated COOP graphically defined: $\vec{p}$ and $\vec{q}$ are completely independent of each other, thus a given $\vec{p}$ does not place any limits on the possible directions of $\vec{q}$; (F) Schematic example of two fields that are anti-correlated—parallel at the ends of the rectangle, yet perpendicular in the middle; (G) The correlated COOP graphically defined: for any $\vec{p}$, there exists a range of angles within which the $\vec{q}$ will be positioned; (H) The ultra-correlated case is similar to the correlated case, but there is also a global organization where the vectors are co-localized maximal angle to minimal angle; (I) Ranges of COOP and Normalized COOP defined on the top and bottom of the bar, respectively. The values of the uncorrelated and correlated COOP limits (sliders on image) are fully defined by the values of OOP_P_ and OOP_Q_. If the COOP_u_>0, the region between zero and COOP_u_ corresponds to the anti-correlated arrangements. If COOP_c_<1, the region between COOP_c_ and one corresponds to the ultra-correlated arrangements; (J)(i) and (ii) COOP_u_ and COOP_c_ as a function of the OOP_P_ and OOP_Q_ respectively; (iii) Maximal allowable error in the OOP for there to be a statistically significant (p<0.05) difference between COOP_u_ and COOP_c_ if the sample size N = 4. (iv) The minimum sample size, for statistical significance, with OOP error of *σ*_*OOP*_ = 0.04.

One of the main goals of this work was to develop a metric to quantify the correlation between the orientation of different biological constructs within the cell or tissue. In designing the new metric, we aimed to overcome the challenge of analyzing orientation of multiple pseudo vectors, i.e. the metric needed to be symmetric with the period of *π* for both vectors. The OOP was designed to analyze the organization of pseudo vectors, and has become a standard parameter for use in liquid crystals [20]. The OOP ranges from zero, for isotropic, to one, for aligned mediums (S1 Fig.), and it has been applied to various biological systems [14, 16, 20]. However, the OOP was not designed to evaluate the correlation of orientation of coupled constructs such as actin fibrils and Z-lines. The first step in creating the COOP was to formally define the problem. Let the first construct be *P*, a set of pseudo vectors $\vec{p_{i}}$, and the second construct be *Q*, a set of pseudo vectors $\vec{q_{i}}$ (Fig. 1A). The order tensor and OOP of each field is:

$$\begin{array}{c} {𝕋}_{K}=\left\langle 2\left[\begin{array}{cc} k_{i,x}k_{i,x} & k_{i,x}k_{i,y}\\ k_{i,x}k_{i,y} & k_{i,y}k_{i,y} \end{array}\right]-𝕀\right\rangle =\left\{Mean\;order\;tensor\right\}, \end{array}$$ (1)

$$\begin{array}{c} OOP_{K}=\max\left[\text{eigenvalue}({𝕋}_{K})\right]=\left\{Orientational\;order\;parameter\;of\;K\right\}, \end{array}$$ (2)

where *K* = {*P*, *Q*}, $\vec{k_{i}}=\{\vec{p_{i}},\vec{q_{i}}\}$, and 𝕀 is the identity matrix. To construct the new metric, we defined a new field *F* (a set of pseudo vectors $\vec{f_{i}}$):

$$\begin{array}{c} f_{i,x}=\vec{p_{i}}\cdot \vec{q_{i}}=p_{i,x}q_{i,x}+p_{i,y}q_{i,y}=\cos\left(\theta \right)\;, \end{array}$$ (3)

$$\begin{array}{c} f_{i,y}=|\vec{p_{i}}\times \vec{q_{i}}|=p_{i,x}q_{i,y}-p_{i,y}q_{i,x}=\sin\left(\theta \right). \end{array}$$ (4)

Physically, field *F* represent the angle (*θ*) between the two biological constructs, $\vec{p_{i}}$ and $\vec{q_{i}}$. The metric was then calculated similarly to the OOP:

$$\begin{array}{c} {𝕋}_{PQ}=\left\langle 2\left[\begin{array}{cc} f_{i,x}f_{i,x} & f_{i,x}f_{i,y}\\ f_{i,x}f_{i,y} & f_{i,y}f_{i,y} \end{array}\right]-𝕀\right\rangle =\left\{Mean\;tensor\;of\;the\;system\right\}. \end{array}$$ (5)

$$\begin{array}{ccc} COOP_{PQ}=\max\left[\text{eigenvalue}({𝕋}_{PQ})\right] & = & \left\{Co\text{-}orientational\;order\right.\\ & & \left.\;parameter\;of\;the\;system\right\}. \end{array}$$ (6)

The analytical solution of the COOP is:

$$\begin{array}{c} COOP_{PQ}=\left\langle 2{\left\{\vec{f_{i}}\cdot \hat{n}\right\}}^{2}-1\right\rangle =\left\langle 2{\left\{\vec{f_{i}}\cdot \hat{n}\right\}}^{2}\right\rangle -1=2\left\langle {\left\{\vec{f_{i}}\cdot \hat{n}\right\}}^{2}\right\rangle -1, \end{array}$$ (7)

where $\hat{n}$, the director, is the eigenvector associated with the maximum eigenvalue of mean tensor 𝕋_*PQ*_. The director represents the mean angle between the two constructs. Alternatively, the COOP can be written in the expended form:

$$\begin{array}{c} COOP_{PQ}=\left\langle f_{i,x}^{2}\right\rangle +\left\langle f_{i,y}^{2}\right\rangle -1+\sqrt{{\left(\left\langle f_{i,x}^{2}\right\rangle -\left\langle f_{i,y}^{2}\right\rangle \right)}^{2}+4{\left\langle f_{i,x}f_{i,y}\right\rangle }^{2}}. \end{array}$$ (8)

The COOP was designed to range between zero and one. Here we present a series of theorems that illustrate the various properties of the COOP.

**Theorem 1: Demonstration of COOP symmetry**. Symmetry is an important characteristic of both the OOP and the COOP because it alleviates calculation errors that may arise when there is a random choice of signs for the pseudo vectors (Fig. 1B). Symmetry can be easily shown for OOP (S1 Supplemental Text). As can be seen from the anyaltical solution of the COOP (Equation 7), it is only necessary to demonstrate the symmetry of ${\left\{\vec{f_{i}}\cdot \hat{n}\right\}}^{2}$ in *π* to demonstrate the pseudo-symmetry of the COOP.

Table 1 shows the eight possible symmetry permutations. All of these can be reduced to the same equation with no difference in sign, which proves pseudo-vector symmetry of the COOP. We also demonstrated that the COOP is symmetric to the switch of *P* and *Q*:

$$\begin{array}{c} f_{i,x}^{\prime }=q_{i,x}p_{i,x}+q_{i,y}p_{i,y}=f_{i,x}\;\text{and}\;f_{i,y}^{\prime }=q_{i,x}p_{i,y}-q_{i,y}p_{i,x}=-f_{i,y}, \end{array}$$ (9)

$$\begin{array}{c} COOP_{QP}=\left\langle f_{x}^{2}\right\rangle +\left\langle {\left(-f_{y}\right)}^{2}\right\rangle -1+\sqrt{{\left(\left\langle f_{x}^{2}\right\rangle -\left\langle {\left(-f_{y}\right)}^{2}\right\rangle \right)}^{2}+4{\left\langle -f_{x}f_{y}\right\rangle }^{2}}=COOP_{PQ}. \end{array}$$ (10)

Symmetry also plays an important role in interpreting the COOP director. There are four valid results for an angles between two pseudo-vectors: *θ*_0_, −*θ*_0_, *π*−*θ*_0_, *θ*_0_−*π* (Fig. 1B). For any symmetry permutation the director will correspond to one of these four angles. However, it is essential that the translation from $\hat{n}$ to *θ*_0_ is handled with this symmetry in mind.

**Table 1:** Symmetry permutations.

$\vec{p_{i}}$	$\vec{q_{i}}$	$\hat{n}$	${\left\{\vec{f_{i}}\cdot \hat{n}\right\}}^{2}$
+	+	+	{(*p*_*i*,*x*_ *q*_*i*,*x*_+*p*_*i*,*y*_ *q*_*i*,*y*_)*n*_*x*_+(*p*_*i*,*x*_ *q*_*i*,*y*_−*p*_*i*,*y*_ *q*_*i*,*x*_)*n*_*y*_}^2^ =
+	+	-	{(*p*_*i*,*x*_ *q*_*i*,*x*_+*p*_*i*,*y*_ *q*_*i*,*y*_)(−*n*_*x*_)+(*p*_*i*,*x*_ *q*_*i*,*y*_−*p*_*i*,*y*_ *q*_*i*,*x*_)(−*n*_*y*_)}^2^ =
+	-	+	{−(*p*_*i*,*x*_ *q*_*i*,*x*_+*p*_*i*,*y*_ *q*_*i*,*y*_)*n*_*x*_−(*p*_*i*,*x*_ *q*_*i*,*y*_−*p*_*i*,*y*_ *q*_*i*,*x*_)*n*_*y*_}^2^ =
+	-	-	{−(*p*_*i*,*x*_ *q*_*i*,*x*_+*p*_*i*,*y*_ *q*_*i*,*y*_)(−*n*_*x*_)−(*p*_*i*,*x*_ *q*_*i*,*y*_−*p*_*i*,*y*_ *q*_*i*,*x*_)(−*n*_*y*_)}^2^ =
-	+	+	{−(*p*_*i*,*x*_ *q*_*i*,*x*_+*p*_*i*,*y*_ *q*_*i*,*y*_)*n*_*x*_−(*p*_*i*,*x*_ *q*_*i*,*y*_−*p*_*i*,*y*_ *q*_*i*,*x*_)*n*_*y*_}^2^ =
-	+	-	{−(*p*_*i*,*x*_ *q*_*i*,*x*_+*p*_*i*,*y*_ *q*_*i*,*y*_)(−*n*_*x*_)−(*p*_*i*,*x*_ *q*_*i*,*y*_−*p*_*i*,*y*_ *q*_*i*,*x*_)(−*n*_*y*_)}^2^ =
-	-	+	{(*p*_*i*,*x*_ *q*_*i*,*x*_+*p*_*i*,*y*_ *q*_*i*,*y*_)*n*_*x*_+(*p*_*i*,*x*_ *q*_*i*,*y*_−*p*_*i*,*y*_ *q*_*i*,*x*_)*n*_*y*_}^2^ =
-	-	-	{(*p*_*i*,*x*_ *q*_*i*,*x*_+_*i*,*y*_ *q*_*i*,*y*_)(−*n*_*x*_)+(*p*_*i*,*x*_ *q*_*i*,*y*_−*p*_*i*,*y*_ *q*_*i*,*x*_)(−*n*_*y*_)}^2^ =
Result for All			= {(*p*_*i*,*x*_ *q*_*i*,*x*_+*p*_*i*,*y*_ *q*_*i*,*y*_)*n*_*x*_+(*p*_*i*,*x*_ *q*_*i*,*y*_−*p*_*i*,*y*_ *q*_*i*,*x*_)*n*_*y*_}^2^

**Theorem 2: Field rotation does not affect COOP**. To verify that rotation of *Q* with respect to *P* does not affect COOP (Fig. 1C), let

$$\begin{array}{c} \vec{p_{i}}=\left[\cos(\alpha ),\sin(\alpha )\right]\;\text{and}\;\vec{q_{i}}=\left[\cos(\beta ),\sin(\beta )\right]. \end{array}$$ (11)

If each $\vec{q_{i}}$ was rotated by angle *ν*, the rotated field *Q*_*rot*_ would be defined by:

$$\begin{array}{ccc} {\vec{q}}_{i,rot} & = & \left[\cos(\beta +\nu ),\sin(\beta +\nu )\right]\\ & = & \left[\cos(\beta )\cos(\nu )-\sin(\beta )\sin(\nu ),\sin(\beta )\cos(\nu )+\cos(\beta )\sin(\nu )\right]. \end{array}$$ (12)

Then the rotated field *F*_*rot*_ is:

$$\begin{array}{c} {\vec{f}}_{i,rot}=\left[f_{i,x}\cos\left(\nu \right)-f_{i,y}\sin\left(\nu \right),f_{i,y}\cos\left(\nu \right)+f_{i,x}\sin\left(\nu \right)\right]. \end{array}$$ (13)

As the angle *ν* is constant:

$$\begin{array}{ccc} \left\langle f_{i,rot,x}\right\rangle & = & \left\langle f_{i,x}\right\rangle \cos\left(\nu \right)-\left\langle f_{i,y}\right\rangle \sin\left(\nu \right)\;\text{and}\;\\ \left\langle f_{i,rot,y}\right\rangle & = & \left\langle f_{i,y}\right\rangle \cos\left(\nu \right)+\left\langle f_{i,x}\right\rangle \sin\left(\nu \right). \end{array}$$ (14)

In combining Equation (13) and (8), all terms with *ν* cancel or are reduced to cos^2^(*ν*)+sin^2^(*ν*) = 1. As a result *COOP*_*rot*_ = COOP. Thus we have proven that field rotation does not affect COOP, and without loosing generality we can assume ${\hat{n}}_{p}={\hat{n}}_{q}=[1,0]$. This proves that the mean angle between fibers cannot be used to evaluate the correlation of orientations.

**Theorem 3: The same field compared to itself gives COOP of 1**. We next proved that the same field compared to itself would obtain a COOP of one (Fig. 1D). Imagine two sets of pseudo vectors distributed in a 2D space, $\vec{p_{i}}$ and $\vec{q_{i}}$. Let

$$\begin{array}{c} \vec{p_{i}}=\left[\cos(\alpha ),\sin(\alpha )\right],\;\text{and}\;\vec{q_{i}}=\left[\cos(\alpha ),\sin(\alpha )\right]. \end{array}$$ (15)

Then, the field $\vec{f_{i}}$ of this system can be written as:

$$\begin{array}{c} f_{i,x}=p_{i,x}q_{i,x}+p_{i,y}q_{i,y}=\cos^{2}\left(\alpha \right)+\sin^{2}\left(\alpha \right)=1\;\text{and} \end{array}$$ (16)

$$\begin{array}{c} f_{i,y}=p_{i,x}q_{i,y}-p_{i,y}q_{i,x}=\cos\left(\alpha \right)\sin\left(\alpha \right)-\sin\left(\alpha \right)\cos\left(\alpha \right)=0. \end{array}$$ (17)

The mean tensor:

$$\begin{array}{c} {𝕋}_{PQ}=\left\langle 2\left[\begin{array}{cc} f_{i,x}f_{i,x} & f_{i,x}f_{i,y}\\ f_{i,x}f_{i,y} & f_{i,y}f_{i,y} \end{array}\right]-𝕀\right\rangle =\left\langle 2\left[\begin{array}{cc} 1 & 0\\ 0 & 0 \end{array}\right]-𝕀\right\rangle =\left[\begin{array}{cc} 1 & 0\\ 0 & -1 \end{array}\right]. \end{array}$$ (18)

Therefore, the COOP of constructs *P* and *Q*:

$$\begin{array}{c} COOP_{PQ}=\max\left[\text{eigenvalue}\left(\left[\begin{array}{cc} 1 & 0\\ 0 & -1 \end{array}\right]\right)\right]=1. \end{array}$$ (19)

We obtained a COOP of 1 thus constructs *P* and *Q* are a perfectly co-oriented system which is expected as *P* = *Q*.

**Theorem 4: Uncorrelated COOP limit**. For any given pair of fields with OOP_P_ and OOP_Q_ there is a range of possible COOP values, with a maximum range of zero to one. To aid in interpreting the meaning of the COOP, we derived the value for the COOP (COOP_u_) as a function of the OOPs for the maximally uncorrelated system. For the COOP to be uncorrelated the two fields needed to have no correlation between their orientations (Fig. 1E). If and only if $\vec{p_{i}}$ and $\vec{q_{i}}$ are assumed independent and ${\hat{n}}_{p}={\hat{n}}_{q}$ (Theorem 2), the analytical solution Equation (7) can be re-written as:

$$\begin{array}{c} COOP_{u}=\left\langle 2{\left(p_{i,x}q_{i,x}+p_{i,y}q_{i,y}\right)}^{2}-1\right\rangle =\left\langle \left(2p_{i,x}^{2}-1\right)\left(2q_{i,x}^{2}-1\right)\right\rangle . \end{array}$$ (20)

Using the classical probability distribution property:

$$\begin{array}{c} COOP_{u}=\left\langle \left(2p_{i,x}^{2}-1\right)\left(2q_{i,x}^{2}-1\right)\right\rangle =\left\langle (2p_{i,x}^{2}-1)\right\rangle \left\langle (2q_{i,x}^{2}-1)\right\rangle . \end{array}$$ (21)

Based on the definition of the OOP the uncorrelated COOP is:

$$\begin{array}{c} COOP_{u}=\left\langle (2p_{i,x}^{2}-1)\right\rangle \left\langle (2q_{i,x}^{2}-1)\right\rangle =OOP_{P}OOP_{Q}=\left\{Uncorrelated\;COOP\right\}. \end{array}$$ (22)

COOP_u_ is the lowest limit of the COOP if and only if the two constructs are independent. However, if *P* and *Q* are correlated in multiple ways it is possible to achieve a smaller value of COOP.

**Theorem 5: Anti-correlated COOP**. To prove that COOP_u_ is not necessarily the minimum COOP, we constructed an example where *P* was composed of $\vec{p_{1}}$ and $\vec{p_{2}}$, and construct *Q* was composed of $\vec{q_{1}}$ and $\vec{q_{2}}$:

$$\begin{array}{c} \vec{p_{1}}=\left[\cos(\alpha ),\sin(\alpha )\right]\;\text{and}\;\vec{p_{2}}=\left[\cos(-\alpha ),\sin(-\alpha )\right]. \end{array}$$ (23)

$$\begin{array}{c} \vec{q_{1}}=\left[\cos\left(\alpha +\frac{\pi }{2}\right),\sin\left(\alpha +\frac{\pi }{2}\right)\right]\;\text{and}\;\vec{q_{2}}=\left[\cos(-\alpha ),\sin(-\alpha )\right]. \end{array}$$ (24)

Construct *P* has an *OOP*_P_ = cos(2*α*) and construct *Q* has an *OOP*_Q_ = ∣sin(2*α*)∣. The vector $\vec{f}$ is:

$$\begin{array}{c} f_{1,x}=0\;,\;f_{2,x}=1\;\text{and}\;f_{1,y}=1\;,\;f_{2,y}=0. \end{array}$$ (25)

The mean tensor and COOP of this system are:

$$\begin{array}{c} {𝕋}_{PQ}=\left[\begin{array}{cc} 0 & 0\\ 0 & 0 \end{array}\right]\;\text{and}\;COOP=0. \end{array}$$ (26)

Using Equation (22) we obtained:

$$\begin{array}{c} COOP_{u}=OOP_{P}\cdot OOP_{Q}=\cos\left(2\alpha \right)\left|\sin(2\alpha )\right|. \end{array}$$ (27)

Unless, $\alpha \neq \{0,\frac{\pi }{4},\frac{\pi }{2}\}\cdot n$ for *n* = {1, 2, …}, COOP_u_>0. Thus, the COOP can be lower than COOP_u_. Such a case can be graphically imagined if you have constructs correlated in two different ways in the same cell (Fig. 1F), similar to a single cardiomyocyte with punctate alpha-actinin at the ends and well defined Z-lines in the middle. This demonstrates that, similarly to the OOP, the COOP will not fully capture second order correlations (S1 Supplemental Text).

**Theorem 6: Correlated COOP limit**. For the upper limit, we derived the value of the COOP as a function of the OOPs for the maximally correlated constructs. To determine the correlated COOP (COOP_c_), we assumed the two fields, *P* and *Q*, were almost identical, except that *Q* was rotated by a random noise angle *θ* (Fig. 1G). We can assume, without loosing generality, ${\hat{n}}_{p}={\hat{n}}_{q}=[1,0]$ (Theorem 2), and *P* is better organized (i.e. OOP_P_>OOP_Q_, Theorem 1). Using the analytical solutions, the OOP_P_ and COOP were rewritten as:

$$\begin{array}{c} OOP_{P}=\left\langle 2\cos^{2}\left(\alpha \right)-1\right\rangle \;\text{and}\;COOP=\left\langle 2\cos^{2}\left(\theta \right)-1\right\rangle . \end{array}$$ (28)

As *θ* was assumed to be random noise generated, *θ* and *α* are independent, and therefore:

$$\begin{array}{ccc} OOP_{Q} & = & \left\langle 2\cos^{2}\left(\alpha -\theta \right)-1\right\rangle \\ & = & \left\langle \left(2\cos^{2}\left(\alpha \right)-1\right)\left(2\cos^{2}\left(\theta \right)-1\right)+4\cos\left(\alpha \right)\sin\left(\alpha \right)\cos\left(\theta \right)\sin\left(\theta \right)\right\rangle \\ & = & \left\langle \left(2\cos^{2}\left(\alpha \right)-1\right)\left(2\cos^{2}\left(\theta \right)-1\right)\right\rangle +\left\langle 2\cos(\alpha )\sin(\alpha )\right\rangle \left\langle 2\cos(\theta )\sin(\theta )\right\rangle \\ & = & \left\langle (2\cos^{2}\left(\alpha \right)-1)\right\rangle \left\langle (2\cos^{2}\left(\theta \right)-1)\right\rangle +\left\langle \sin(2\alpha )\right\rangle \left\langle \sin(2\theta )\right\rangle \\ & = & OOP_{P}COOP_{c}. \end{array}$$ (29)

Solving for COOP_c_ and rewriting it in a more general form we obtained:

$$\begin{array}{c} COOP_{c}=\frac{min(OOP_{P},OOP_{Q})}{max(OOP_{P},OOP_{Q})}. \end{array}$$ (30)

This is the upper limit of COOP if the two constructs are correlated but are subject to random biological variance (noise). This would not be the limit in a system where the variance is not random.

**Theorem 7: Ultra-correlated COOP**. We also showed that the correlated COOP is not necessarily the maximum. To prove this we defined *P* and *Q* as:

$$\begin{array}{c} \vec{p_{i}}=\left[\cos\left(\alpha_{i}\right),\sin\left(\alpha_{i}\right)\right]\;\text{and}\;{\vec{p}}_{n+i}=\left[\cos\left(-\alpha_{i}\right),\sin\left(-\alpha_{i}\right)\right] \end{array}$$ (31)

$$\begin{array}{c} \vec{q_{i}}=\left[\cos\left(\alpha_{i}+\theta_{i}\right),\sin\left(\alpha_{i}+\theta_{i}\right)\right]\;\text{and}\;{\vec{q}}_{n+i}=\left[\cos\left(-\alpha_{i}-\theta_{i}\right),\sin\left(-\alpha_{i}-\theta_{i}\right)\right] \end{array}$$ (32)

for *i* = {1, …, *n*}. Thus, each set of angles is paired in decreasing order (Fig. 1H). OOP_P_ and OOP_Q_ are defined as:

$$\begin{array}{c} OOP_{P}=\frac{1}{n}\sum_{i=1}^{n}\cos\left(2\alpha_{i}\right)\;\text{where}\;\alpha_{i}:\sum_{i=1}^{n}\cos\left(2\alpha_{i}\right)\geq 0 \end{array}$$ (33)

$$\begin{array}{c} OOP_{Q}=\frac{1}{n}\sum_{i=1}^{n}\cos\left(2\alpha_{i}+2\theta_{i}\right)\;\text{where}\;\alpha_{i},\theta_{i}:\sum_{i=1}^{n}\cos\left(2\alpha_{i}+2\theta_{i}\right)\geq 0. \end{array}$$ (34)

Assuming $\sum_{i=1}^{n}\cos(2\theta_{i})\geq 0$ the mean tensor is:

$$\begin{array}{c} {𝕋}_{PQ}=\left[\begin{array}{cc} \frac{2}{n}\sum_{i=1}^{n}\cos^{2}\left(\theta_{i}\right) & 0\\ 0 & -\frac{1}{n}\sum_{i=1}^{n}\left(2\cos^{2}\left(\theta_{i}\right)-2\right) \end{array}\right]. \end{array}$$ (35)

Knowing $COOP=\frac{1}{n}\sum_{i=1}^{n}\cos(2\theta_{i})$ and the ${\text{COOP}}_{\text{c}}=\frac{OOP_{\text{Q}}}{OOP_{\text{P}}}$:

$$\begin{array}{c} COOP-COOP_{c}=\frac{1}{n}\sum_{i=1}^{n}\cos\left(2\theta_{i}\right)-\frac{\sum_{i=1}^{n}\cos\left(2\alpha_{i}+2\theta_{i}\right)}{\sum_{i=1}^{n}\cos\left(2\alpha_{i}\right)}. \end{array}$$ (36)

If the expression in Equation (36) is positive, then *COOP* ≥ *COOP*_c_. An example of such a case is when *θ* is constant and Equation (36) becomes:

$$\begin{array}{c} COOP-COOP_{c}=\frac{\sum_{i=1}^{n}\sin\left(2\alpha_{i}\right)\sin\left(2\theta_{i}\right)}{\sum_{i=1}^{n}\cos\left(2\alpha_{i}\right)}. \end{array}$$ (37)

Based on conditions in Equation (33) and (34), $0\leq \alpha_{i}\leq \frac{\pi }{2}$ and $0\leq \theta_{i}\leq \frac{\pi }{2}$, which implies sin(2*α*_*i*_) ≥ 0, cos(2*α*_*i*_) ≥ 0, and sin(2*θ*_*i*_) ≥ 0 for all *i*. Therefore, every term in Equation (37) is positive. If *P* is not perfectly aligned (i.e. *α*_*i*_ ≠ 0), and if *P* and *Q* are not identical (i.e. *θ*_*i*_ ≠ 0), then COOP>COOP_c_. Thus, there is an ultra-correlated COOP that can be greater than *COOP*_c_. Qualitatively, the ultra-correlated system is similar to the correlated example, except the $\vec{q}$ vectors are not random within the noise, but are arranged maximum to minimum angles (Fig. 1H).

**Normalized COOP**. Based on Theorems 4–7 we can divide the range of COOP into three regions (Fig. 1I): anti-correlated, normal, and ultra-correlated. The boundaries of these regions (COOP_u_ and COOP_c_) are determined by the organization of the two constructs (OOP_p_ and OOP_q_) and will slide along the overall range [0, 1]. Experimentally, it might be more relevant to know how close the COOP is to the uncorrelated and correlated boundaries as these carry biological implications. We therefore defined a Normalized COOP, which is a measure of how close a parameter is to COOP_u_ or COOP_c_:

$$\begin{array}{c} \text{Normalized}\;\text{COOP}=\frac{COOP-COOP_{u}}{COOP_{c}-COOP_{u}}. \end{array}$$ (38)

The normalized COOP is negative when it is anti-correlated, zero when the system is uncorrelated, one when it is correlated, and greater than one when it is ultra-correlated (Fig. 1I). Any parameter has limitations. For example, the OOP of the red line-segments in the anti-correlated schematic (Fig. 1F) would be equivalent to an isotropic organization (*OOP* ∼ 0) even though the red line-segments are well organized. Similarly, the COOP cannot be used to identify correlations in tissues that have very non-trivial spatially dependent correlations (more complex than the ultra-correlated case (Fig. 1H)). We believe that most biological constructs for which the COOP has been developed will never exhibit this behavior. However, if the COOP is ever found to be statistically significantly greater than COOP_c_, it will be essential to re-evaluate the applicability of the parameter.

**Estimated maximum tolerable error and minimum sample size**. To interpret the information provided by the COOP, we would need to know which region our tissue falls under. Our ability to do so will be limited by the error inherent in any measurement and the width of the normal COOP range. COOP_u_ approaches the maximum (COOP_u_ = 1) as both OOP_p_ and OOP_q_ approach one (Fig. 1J(i)). COOP_c_, however, approaches its maximum when the two order parameters are close to being equal (Fig. 1J(ii)). This shows, for example, that if OOP_p_ = 0 and OOP_q_ = 0, then the normal region of the COOP ranges [0, 1]. However, if OOP_p_>0 and OOP_q_ = 0, the normal range does not exist and a COOP>0 would indicate an ultra-correlated system. If the error in the system is so large that there is no statistically significant difference between the boundaries (COOP_u_ and COOP_c_), it would not be possible to differentiate between the regions. Therefore, for the parameter to be useful, the maximum allowable error and minimum sample size have to be experimentally realistic. To estimate the error and sample size, we calculated the propagation of error in COOP_u_ and COOP_c_, and used them in the student t-test to calculate statistical significance.

Assuming OOP_P_ and OOP_Q_ are normally distributed with the standard deviation *σ*_*OOP*_P__ and *σ*_*OOP*_Q__, respectively, and OOP_P_ and OOP_Q_ are independent, then the variance:

$$\begin{array}{ccc} \sigma_{COOP_{u}}^{2} & = & {\left(\frac{\partial COOP_{u}}{\partial OOP_{P}}\right)}^{2}\sigma_{OOP_{P}}^{2}+{\left(\frac{\partial COOP_{u}}{\partial OOP_{Q}}\right)}^{2}\sigma_{OOP_{Q}}^{2}\\ & = & OOP_{Q}^{2}\sigma_{OOP_{P}}^{2}+OOP_{P}^{2}\sigma_{OOP_{Q}}^{2}, \end{array}$$ (39)

and the standard deviation:

$$\begin{array}{c} \sigma_{COOP_{u}}=\sqrt{OOP_{Q}^{2}\sigma_{OOP_{P}}^{2}+OOP_{P}^{2}\sigma_{OOP_{Q}}^{2}}. \end{array}$$ (40)

Assuming OOP_P_>OOP_Q_, the variance for the correlated COOP:

$$\begin{array}{ccc} \sigma_{COOP_{c}}^{2} & = & {\left(\frac{\partial COOP_{c}}{\partial OOP_{P}}\right)}^{2}\sigma_{OOP_{P}}^{2}+{\left(\frac{\partial COOP_{c}}{\partial OOP_{Q}}\right)}^{2}\sigma_{OOP_{Q}}^{2}\\ & = & {\left(\frac{OOP_{Q}}{OOP_{P}^{2}}\right)}^{2}\sigma_{OOP_{P}}^{2}+{\left(\frac{1}{OOP_{P}}\right)}^{2}\sigma_{OOP_{Q}}^{2}. \end{array}$$ (41)

Note that $\frac{\partial COOP_{\text{c}}}{\partial OOP_{\text{P}}}$ and ${\frac{\partial COOP_{\text{c}}}{\partial OOP_{\text{Q}}}}^{2}$ do not exist when OOP_P_ = OOP_Q_. However, using Theorem 1, we formulated the estimate for the the standard deviation of COOP_c_:

$$\begin{array}{c} \sigma_{COOP_{c}}=\left\{\begin{array}{cc} \sqrt{{\left(\frac{OOP_{Q}}{OOP_{P}^{2}}\right)}^{2}\sigma_{OOP_{P}}^{2}+{\left(\frac{1}{OOP_{P}}\right)}^{2}\sigma_{OOP_{Q}}^{2}} & \text{:}\;OOP_{P}>OOP_{Q}\\ \sqrt{{\left(\frac{1}{OOP_{Q}}\right)}^{2}\sigma_{OOP_{P}}^{2}+{\left(\frac{OOP_{P}}{OOP_{Q}^{2}}\right)}^{2}\sigma_{OOP_{Q}}^{2}} & \text{:}\;OOP_{P}<OOP_{Q}. \end{array}\right. \end{array}$$ (42)

Then to estimate maximum allowable error, we assumed *σ*_*OOP*_P__ = *σ*_*OOP*_Q__, and tested the null hypothesis of COOP_u_ = COOP_c_. We calculated the t-value and degrees of freedom to complete the t-test. We assumed significance for a p-value less than 0.05 for the two sample t-test. There are two variables that impact significance as a function of OOPs: the error (*σ*_*OOP*_) and the sample size (N). For visualization, we calculated the maximum allowable experimental error (*max*(*σ*_*OOP*_)) at sample size of four (Fig. 1J(iii)). The maximal allowable error at N = 4 was at its highest, *max*(*σ*_*OOP*_) = 0.18, for OOP_P_ = OOP_Q_ = 0.60, which is greater than experimental error reported for OOP organization [13]. While the maximal allowable error approaches zero as OOP→0, it rapidly increases for OOP>0.

We also determined the minimum sample size that provides statistical significance (p<0.05) as a function of construct organization for *σ*_*OOP*_ = 0.04. The minimum sample size ranges from two to infinity (Fig. 1J(iv)). Naturally, when COOP_c_→COOP_u_, the *min*(*N*) → ∞ as it is not possible to find them significantly different. For *σ*_*OOP*_ = 0.04, the minimum sample size is between two and five for most of the OOP values. For convenience, we also calculated the minimum sample size for a range of higher experimental errors (S2 Fig.). Obviously higher error would necessitate more samples to maintain significance. However, errors reported for OOP experimentally correspond to normal requirements in sample size. The errors and sample sizes were confirmed to be experimentally realistic, thus we next moved to testing the parameter with synthetic and experimental data.

### Synthetic Results

**Fig 2:**
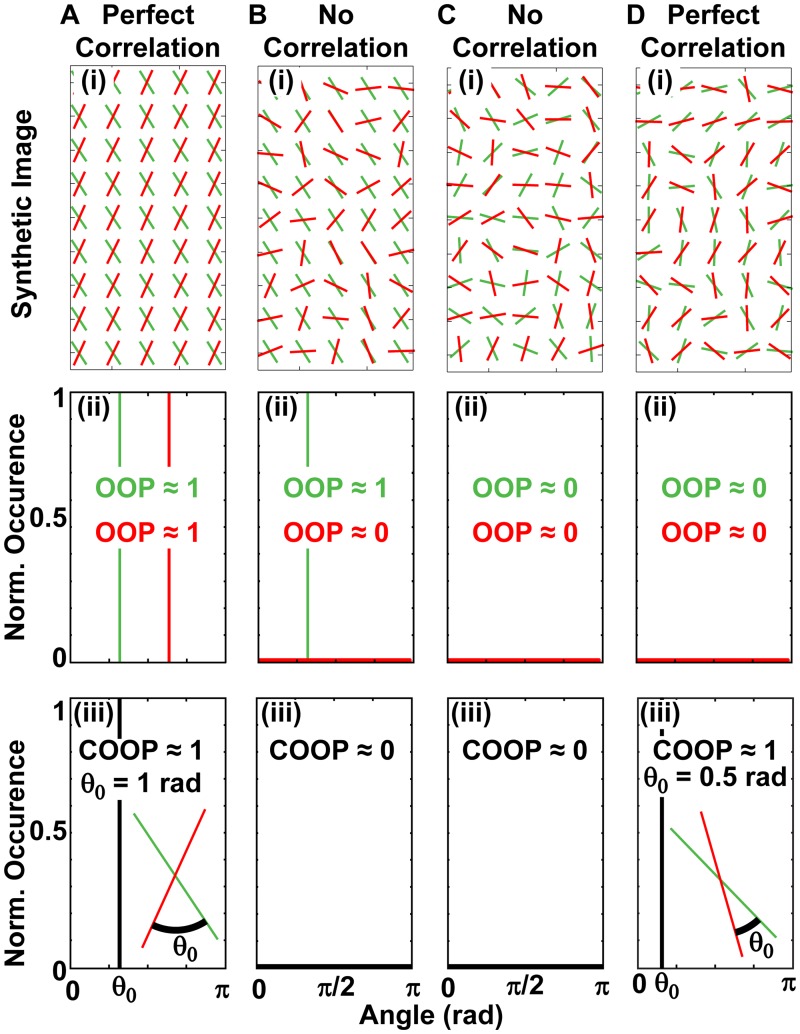
Synthetic data for four limiting cases. A) Two perfectly organized samples are always perfectly correlated; (B) A perfectly organized and an isotropic construct cannot be correlated; Two isotropic cases can be (C) completely uncorrelated and (D) perfectly correlated. For (A-D) (i) Schematic of a small section of the synthetic data; (ii) OOP and normalized occurrence for both constructs; (iii) COOP results with *θ*_0_ designating the mean angle between constructs.

**Limiting cases**. The first step to validate the new parameter was to construct four limiting cases that should lead to specific COOP values. We constructed a custom MatLab code that could be interfaced with experimental or synthetic (computer generated) data. The code was used to confirm the COOP for four synthetic limiting cases. The first test was to compare two perfectly aligned constructs (Fig. 2A(i)). It was clear from the image that these two constructs were perfectly correlated, and we therefore expected COOP = 1. We first confirmed that both constructs were perfectly aligned with OOP_P_ = OOP_Q_ = 1 (Fig. 2A(ii)). Analytically, it is clear that COOP = 1, by first applying Theorem 2 and then Theorem 3. When the synthetic data for this condition was analyzed by the code, the COOP was confirmed to be one (Fig. 2A(iii)). It is worthwhile to note that the director generated by the COOP code corresponds to the angle, *θ*_0_, between the constructs.

For the second limiting condition, one construct was perfectly organized, while the second was completely disorganized (Fig. 2B(i)). In this case, there was no correlation, and we expected COOP = 0. Analytically, even if we supposed that the two constructs were maximally correlated (COOP = COOP_c_) and applied Theorem 6 based on OOP_Q_ = 1 and OOP_P_ = 0 (Fig. 2B(ii)), the COOP = 0. This was confirmed by the result of the synthetic case (Fig. 2B(iii)).

The third case considered two isotropic constructs that are completely uncorrelated (Fig. 2C(i)-(ii)). We expected the COOP for uncorrelated constructs to be zero, and analytically this was confirmed by Theorem 4. These findings were also confirmed by the results of the synthetic data (Fig. 2C(iii)).

The fourth, most intriguing case, was of two isotropic constructs, which were perfectly correlated (Fig. 2D(i)-(ii)). We expected the COOP to be one as there was perfect correlation, and this was proven by Theorem 6. Synthetically we showed the COOP = 1, and again the director gave the average angle, *θ*_0_, between constructs. The limiting conditions validated the parameter for four simple cases. However, the interpretation of the COOP gets more complex when neither construct is perfectly aligned or isotropic.

**Fig 3:**
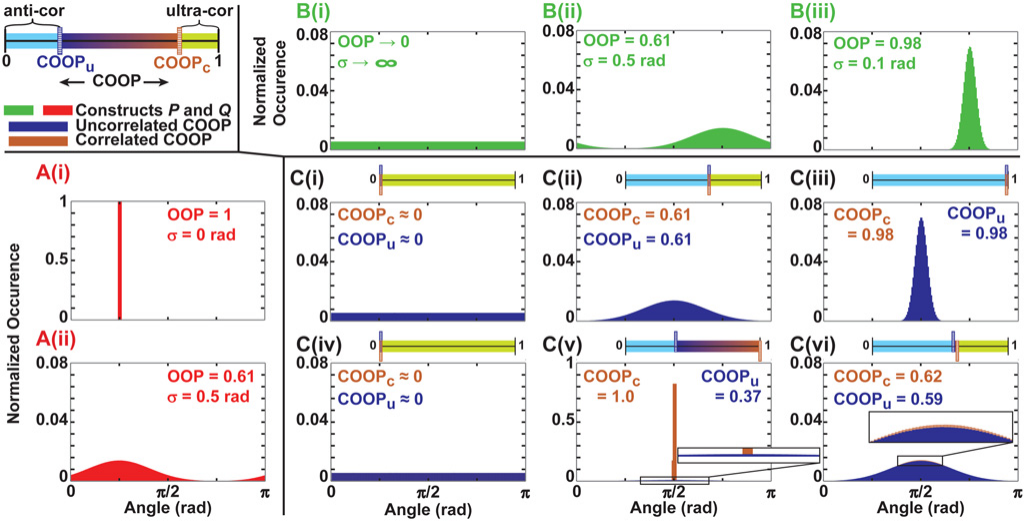
Boundaries of normal region of the COOP. (A and B) The orientation distribution for construct A and B respectively with both the standard deviation of the truncated Gaussian distribution and OOP indicated for each case; (C) the distribution of the angles between the two constructs if they are independent of each other (dark blue) and if the difference is dictated by a random noise (brown). For each case the boundaries, COOP_u_ and COOP_c_, are indicated. Sliders show this graphically with regions colored according to the legend.

**COOP_u_ and COOP_c_ demonstrated with synthetic data**. To understand the limits of the COOP parameter, we constructed a series of cases with different organizations by truncation of Gaussian distributions with specified standard deviations (Fig. 3A and 3B). We created synthetic data for the uncorrelated case (dark blue Fig. 3C) by generating two separate random number sets using the appropriate truncated Gaussian distribution for each. For the correlated case (brown Fig. 3C), we generated the first, more organized, data set by the same method. Then the noise was generated such that when it was added to the first data set, the new set would have the target distribution. Both methods of creating *P* and *Q* data sets lead to the same desired individual distributions (Fig. 3A and 3B). To visualize the results we constructed a slider, sketched in Fig. 1I, for each case (Fig. 3C) color-coded to indicate the boundaries, COOP_u_ (dark blue) and COOP_c_ (brown), as well as the three regions: anti-correlated (light blue), normal (ranging from dark blue to brown), and ultra-correlated (bright green). We expected that when OOP_P_ = 1 (Fig. 3A(i)), COOP_u_ = COOP_c_ = OOP_Q_ for all OOP_Q_ values, which was confirmed by the results of the synthetic data (Fig. 3C(i)-(iii)). Also, if one OOP = 0 (Fig. 3B(i)) and the other *OOP* ≠ 0, the COOP_u_ = COOP_c_ = 0. This was also confirmed with synthetic results (Fig. 3C(i) and 3C(iv)). For a case where *OOP* ≠ {0 or 1} the normal range of the COOP was greater if the OOPs were smaller and closer to each other, which can be seen by comparing Fig. 3C(v) and Fig. 3C(vi). These synthetic results confirm the results of Fig. 1, which showed that the COOP parameter is best applied in situations where the correlation is not obviously dictated by the organization of the individual constructs.

### Experimental Results

**Fig 4:**
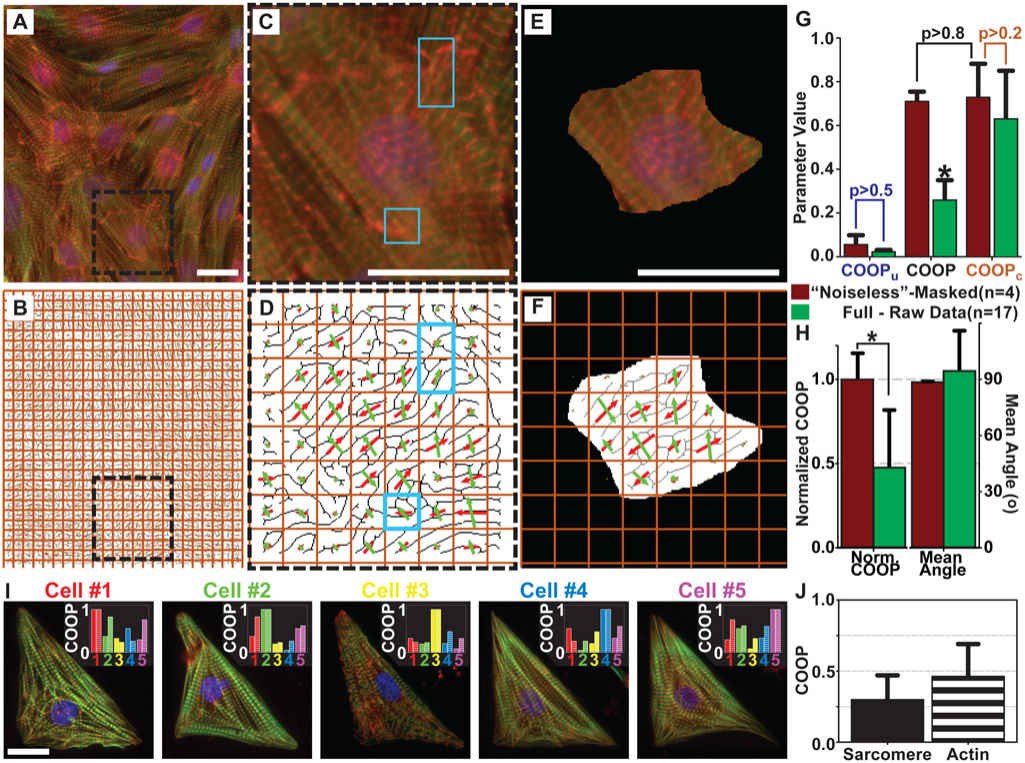
Experimental applications of the COOP. (A) Stained isotropic monolayer of NRVMs; (B) the grid used to calculate average Z-line and actin fibrils direction overlaid on the Z-line skeleton. (C) A section of the stained isotropic NRVM monolayer shown at a higher magnification (corresponds to the dashed box in A). Blue boxes point to some imperfections in the tissue and/or staining with clumps of *α*-actinin. (D) The skeletonized Z-lines from the image in (C) overlaid with the direction of actin fibrils (green arrows) and sarcomere z disks (red arrows) for every grid-square. Note that arrows in blue boxes are not perpendicular. (E) The image from (C) masked to only show noiseless tissue. (F) The actin fibril and Z-line directions overlaid on the Z-line skeleton from (E). (G) COOP_u_, COOP, and COOP_c_ for noiseless and raw images, (*) indicates statistical significance of p<0.001 between the raw COOP and raw COOP_c_. (H) Normalized COOP and mean angle (clockwise from actin fibril to Z-line) for raw and noiseless images. (I) NRVMs cultured on identical FN islands. Each image contains a histogram of COOP between that cell and all others (solid-Z-line, stripe-actin fibril). (J) The average COOP for consistency of Z-line (solid) and actin fibril (stripe) organization. For (A), (C), (E), and (I) stains are: green—actin, red—*α*-actinin, and blue—nuclei. For (G), (H), and (J), error bars represent standard deviation. The n indicates the number of cover-slips, with at least 10 field of view taken for each. Scale bars = 20μm.

The COOP was designed as a tool to evaluate correlations of orientation in experimental samples. We used actin fibers and sarcomeric Z-lines in NRVMs to validate the parameter and code. The tissues were stained for *α*-actinin and phalloidin to identify Z-line and actin fibril directions, respectively (Fig. 4A). The program we used to identify the direction of the construct was based on a finger-print identification code [6, 25, 26], and it assigned a direction to every non-empty pixel in the image. However, computing correlation of constructs based on individual pixels introduced too many errors. Indeed, most images of cytoskeleton constructs are obtained via immunostaining and imaging. The accuracy of the COOP will be a direct consequence of image quality. If the images are of poor quality (poor contrast, dead cells, etc.), it will not be possible to accurately extract the construct direction data, and thus, the COOP will not be accurate. However, if it is possible to accurately extract directionality data, the COOP can be used. The images we have collected for the proof-of-principle data set are representative of the images normally used to study tissue architecture [6, 20]. The images may be resolvable to different degrees, and image acquisition procedures can introduce inaccuracies at smaller scales. For example, during the collection of this data, it is customary to ensure that each channel results in sharp images of the corresponding construct, such as actin and Z-lines. This sometimes requires focusing on slightly different planes, and as a result, the images of the same field-of-view could be slightly off-set from each other. This along with other imaging inaccuracies lead us to develop a procedure to average out these small errors by calculating the direction of each construct within grid-squares. While any consistent small grid can be used for comparing results across multiple tissues, we recommend picking a grid size that would correspond to a natural biological unit. In this case, the image was partitioned into a grid (Fig. 4B), which was chosen such that at minimum, two Z-lines could be expected to fit within each grid-square, ensuring that at least one “sarcomere” complex is within each square (S3 Fig.). The spacing between Z-lines in NRVM tissues is 1.9–2.1 μm [27], thus we chose a grid size close to 4.2 μm (∼ 30 pixels). For each grid (*i*), we calculated the average direction for both constructs (i.e. Z-lines or actin fibrils) (Fig. 4C). To account for varying densities and partial grid-squares at the edges of the images, the area density (*ρ*) was calculated for each grid-square using the number of non zero angles in the grid-square divided by its area. Each grid-square (*i*) was assigned a weight (*W*_*i*_) based on the OOP and density of constructs (*ρ*):

$$\begin{array}{c} W_{i}=OOP_{P,i}\cdot OOP_{Q,i}\cdot \rho_{P,i}\cdot \rho_{Q,i}. \end{array}$$ (43)

Thus, partial grids with low densities have small weight factors whereas full grids have high weight factors (length of arrows in Fig. 4D). Additionally, grid-squares with better alignment have higher weights than grid-squares with isotropic organization, which prevents loss of consistency.

Z-lines and actin fibers were expected to be perfectly correlated within the sarcomeres of the healthy cardiac muscle tissue. However, histological samples may not be perfect, with some example imperfections identified in the zoomed-in image of Fig. 4C, which correspond to the non-perpendicular arrows identified in Fig. 4D. Note that the lengths of the arrows correspond to the weights assigned to each square, so these imperfections can significantly alter the resulting COOP. To test the parameter, we took four coverslips with 10 fields of view imaged for each and identified noiseless regions with minimal imperfections (Fig. 4E-F). Before any angle detection was done, ImageJ was used to merge fields of view containing Z-lines and actin fibrils, and regions with minimal imperfections were chosen by an experienced user. Only merged images that contained four or more regions of minimal imperfections were used (Fig. 4E). This was done prior to the organizational analysis to eliminate bias. The implementation of the code is summarized in a flow chart (S4 Fig.).

The OOPs for actin fibrils and Z-lines were essentially the same for both the raw and masked images. We measured no significant difference in the values of COOP_u_ or COOP_c_ between the full and noiseless data sets (Fig. 4G). There was also no significant difference between COOP and COOP_c_ for the masked, noiseless images. In contrast there was a significant difference between COOP and COOP_c_ (p<0.001) for the raw images. This is reflected in the Normalized COOP which is 1 for the noiseless images and less than 0.5 for the full data (Fig. 4H). This illustrates that while the parameter is capable of capturing the expected correlation in a sarcomere between actin fibril and Z-line orientation, cardiac tissues may have imperfections that result in a lower value of COOP. The mean angle for the noiseless images also shows the expected perpendicular correlation between orientations of sarcomere Z-lines and actin fibrils with minimal error (Fig. 4H). While, they were also approximately perpendicular in the raw images, the error was greater than for the noiseless images.

The COOP can also be used to evaluate the consistency of construct orientation within cells of the same shape. As an example we seeded NRVMs on triangular islands, and stained the samples for nucleus, Z-lines, actin fibrils, and fibronectin (Fig. 4I). For analysis we ensured that the fibronectin islands lined up for all five cells (S5 Fig.). Then the Z-line (solid bars) or actin fibril (dashed bars) images were compared in a pairwise manner (Tables 2 and 3). This showed an additional experimental confirmation of Theorem 3: COOP = 1 for each cell when it was compared with itself (Fig. 4I). The COOP was calculated for the same grid as in the isotropic images. The results showed that although there is an overall consistency between cells, i.e., myofibrils were bundled along the edges of the triangle, the orientation was not fully consistent at a smaller length scale (grid size). Indeed, the average COOP for ten pair-wise comparisons (bold in Tables 2 and 3) of both Z-lines and actin fibrils is less than 0.5 (Fig. 4K). This demonstrated another potential use of this parameter.

**Table 2:** Pairwise comparison of sarcomeric Z-lines consistency.

*COOP*_*sarc*_	Cell 1	Cell 2	Cell 3	Cell 4	Cell 5
Cell 1	1.00	**0.37**	**0.33**	**0.56**	**0.30**
Cell 2	0.37	1.00	**0.20**	**0.05**	**0.55**
Cell 3	0.33	0.20	1.00	**0.21**	**0.09**
Cell 4	0.56	0.05	0.21	1.00	**0.34**
Cell 5	0.30	0.55	0.09	0.34	1.00

**Table 3:** Pairwise comparison of actin fibrils consistency.

*COOP*_*actin*_	Cell 1	Cell 2	Cell 3	Cell 4	Cell 5
Cell 1	1.00	**0.83**	**0.24**	**0.26**	**0.76**
Cell 2	0.83	1.00	**0.23**	**0.26**	**0.62**
Cell 3	0.24	0.23	1.00	**0.48**	**0.33**
Cell 4	0.26	0.26	0.48	1.00	**0.61**
Cell 5	0.76	0.62	0.33	0.61	1.00

## Discussion

Colocalization, a process that analyzes the spatial overlap between two biological constructs, has been key in discovering cellular mechanisms that rely on the proximity of constructs [28, 29]. For example, second-order stereology has been used to analyze spatial arrangements of constructs in images. Noorafshan et al. used second-order stereology to examine the correlation between the spatial arrangements of cardiomyocytes and microvessels [22]. Their method involved pair correlation and cross-correlation functions to determine positive or negative correlation at different distances. However, neither simple colocalization nor second-order stereology do not analyze the relative orientations of biological constructs.

In this work, we have developed a new parameter, COOP, to characterize how two tissue components align with respect to each other. The COOP would allow for investigation of mechanisms or functions that rely on not only spatial proximity, but also specific organizational schemes. To properly interpret the meaning of the parameter values, we characterized it through a series of analytical theorems. As a result, we defined three regimes—normal, ultra-correlated, and anti-correlated—that have biological implications. After validating the parameter with synthetic data, we demonstrated its use with experimental images by showing that perfect portions of cardiac tissues have the expected correlation of the orientation between sarcomeric z-discs and actin fibrils. The reduction in the COOP for un-masked (raw) data suggests that the defects in the architecture will be distinguished by our new method (Fig. 4G-H). The code we have developed can also be used to calculate the mean angle between constructs thus allowing for tracking of mean angle changes as a function of experimental conditions. Furthermore, the parameter can be used to calculate the consistency of orientational organization to help evaluate the importance of orientational order.

To compare organization between different experimental conditions, it is necessary to have a robust metric. The best metrics place the least number of constraints on the distribution of orientations. For example, the standard deviation is not an appropriate metric for quantifying orientation distribution of Z-lines as they are not distributed normally. The OOP works with all types of distributions, and it has an additional benefit of being symmetric to pseudo-vectors [14]. As the COOP was designed with similar math, it shares the same benefits as the OOP such as pseudo-vector symmetry.

In general, there are multiple ways to use mathematical functions to analyze the properties of images. For example, Feng et al. use normalized cross-correlation to compare two images with a possible rotation or change in scale [23]. Their method involves identifying a relatively small number of points of interest and matching the comparison based on them. The Feng et al. method is insensitive to the rotation of the whole image (i.e. rotation plus translation), while the COOP method is insensitive to the rotation of all vectors without translation. The normalized cross-correlation is a powerful tool, but not appropriate whole cell architectural metric, the COOP is therefore useful for comparing consistency in similarly shaped cells with matching ECM islands, but cannot be used to identify the same cell that has been re-scaled and rotated. Another example of mathematical tools for image analysis is a set of a non-parametric circular statistics tests such as Watson’s $U_{n}^{2}$ test, which is designed to evaluate the probability that a sample comes from a specific distribution or that two samples come from the same distribution. For instance, non-parametric circular statistics has been utilized to evaluate if a pattern of migration of different objects is the same [30, 31]. However, these tests do not consider the location of each sample pair, thus while the results can correspond to the COOP in very special situations these parameters address fundamentally different questions. Thus, these circular statistics tests are not a good tool to evaluate orientational correlation of co-localized pseudo-vectors.

There are specific cases where the COOP will correspond to other parameters. For example, the OOP has been used to quantify the organization of the bacterial population in a channel with respect to the channel direction [16]. Indeed, this is equivalent to a rudimentary case of the COOP where one of the constructs, the channel, is perfectly organized (Fig. 3C(ii)-(iii)). The COOP is more general in that it can be used when neither construct is perfectly organized. Circular statistics tool-sets include some correlation metrics [24], such as the circular correlation coefficient [21] which corresponds to the COOP in the same case. Specificically the circular correlation coefficient can only be used for uniform distributions (i.e. isotropic tissues). In that special case, the circular correlation coefficient and the COOP converge to the same equation (S1 Supplemental Text). However, the more general vector formulation of the circular correlation coefficient, while not constrained to a uniform distribution, is very complex, and thus cannot be easily characterized the way we have done for the COOP. This circular correlation coefficient would not be a convenient metric for cytoskeleton or cellular orientation quantification. The COOP can be calculated so long as two sets of angular distributions and their locations are known, and it has been extensively characterized. Thus, this new parameter can be used with a multitude of biological systems.

In a healthy, properly functioning cell or tissue, the cytoskeleton needs to be organized in an intricate manner. In disease, loss of this organization leads to reduction in function, such as the myofibril organization changes in dilated cardiomyopathy [32–34]. However, proper organization of one element in a cell or tissue is not sufficient. The multiple constructs have to be properly organized with respect to each other, and that organization can have biological implications. This has been shown to occur during maturation of myocytes where the *α*-actinin is initially punctate and parallel to actin fibrils, but, in mature cardiomyocytes, becomes part of the newly formed sarcomeric Z-lines, which are perpendicular to actin fibrils [35]. In this case the relative orientation of *α*-actinin staining and actin fibrils indicates the progressive maturation of myofibrils. An additional example where orientation of different constructs affects each other is when the organization of the extracellular matrix can be used to control the architecture of cells [2, 13, 36]. Conversely, cells have been shown to change the orientation of the extracellular matrix fibrils [37]. Another instance of organization correlation can be found in the different cell types and collagen fibrils within heart valves [38]. The common use of such metrics as the OOP and COOP for biological and medical sciences will allow for a quantitative evaluation of tissue engineered substrates from a variety of cell sources. Combining such metrics with histology will create a universal evaluation metric between *in vitro* and *in vivo* systems favorably impacting our ability to design replacement tissues, to create *in vitro* drug testing platforms, and to evaluate pathological reports in the clinic.

## Materials and Methods

### Ethics Statement

All animals were treated according to the Institutional Animal Care and Use Committee of UCI guidelines (Animal Experimentation Protocol permit number 2013-3093-0). This protocol met the guidelines for the use of vertebrate animals in research and teaching of the Faculty of Arts and Sciences of UCI. It also followed recommendations of the NIH Guide for the Care and Use of Laboratory Animals and was in accordance with existing federal (9 CFR Parts 1, 2 & 3), state, and city laws and regulations governing the use of animals in research and teaching.

### Implementing COOP Calculation

To facilitate the calculation of the COOP we created a custom MATLAB code. The code was designed to have an input of angles for P and Q organized such that the information of which pseudo-vectors are paired was not lost. The code outputs were OOP_P_, OOP_Q_, COOP, COOP_u_, COOP_c_, $\hat{n}$, and *θ*.

### Synthetic Data

Synthetic data of isotropic constructs for limiting conditions (Fig. 2) was generated using a random number generator (rand) that provides a uniform distribution of at least 10^6^ random values in MATLAB. Each construct used in testing COOP_u_ and COOP_c_ (Fig. 3) contained 10^8^ random numbers (MATLAB function normrnd) that were normally distributed with the specified mean and standard deviation. We have included the codes to create synthetic data as supporting codes (S1 Code and S2 Code).

### Experimental Data

**Microcontact printing and ECM patterns**. To make the substrates 25 mm glass coverslips were coated with PDMS (Ellsworth Adhesives, Germantown, WI) and cured for 12 hours in a 60°C oven. To create triangular myocytes we utilized a microcontact printing procedure similar to that described by Tan et al [39]. A mask with the desired pattern was designed using Adobe Illustrator (Adobe Systems Incorporated, San Jose, CA) and made by Front Range Photomask (Palmer Lake, CO). The mask was used to make a silicone wafer (Integrated Nanosystems Research Facility, Irvine, CA). A polydimethylsiloxane (PDMS) stamp, cast from a silicon master, was used to contact transfer the extracellular matrix (ECM) protein fibronectin (FN) (Fisher Scientific Company, Hanover Park, IL) onto a UV-sterilized (UVO, Jelight Company, Inc. Irvine, CA) PDMS-coated coverslip. Fabricated substrates underwent one 10 minute pluronics (250g of Pluronics F-127, Sigma-Aldrich, Inc., Saint Louis, MO) wash and three rinses of phosphate buffer-saline (PBS) (Life Technologies, Carlsbad, CA). To make isotropic substrates, UV-sterilized PDMS-coated coverslip were coated with FN for 10 minutes and underwent three PBS washes. The substrates were stored at 4°C prior to NRVM seeding.

**Cardiomyocyte culture**. Cell cultures of NRVMs were prepared from two-day old Sprague-Dawley rats (Charles River Laboratories, Wilmington, MA). A mid-sternal incision was made in order to expose the heart of the neonatal rat for dissection. Ventricular tissue was removed and rinsed in a Hanks balanced salt solution buffer (Life Technologies, Carlsbad, CA) and placed in 1 mg/mL trypsin solution (Sigma-Aldrich, Inc., Saint Louis, MO) to be shaken overnight (12 hour incubation) at 4°C. The next day, isolated tissue was dissociated into individual cells by treatment with four separate washes of 1 mg/mL collagenase type II (Worthington Biochemical, Lakewood, NJ) for two minutes at 37°C. Isolated cardiomyocytes were resuspended in M199 culture medium (Invitrogen, Carlsbad, CA) supplemented with 10% heat-inactivated Fetal Bovine Serum, 10 mM HEPES, 20 mM glucose, 2 mM L-glutamine, 1.5 μM vitamin B-12 and 50 U/ml penicillin. The cell solution was filtered with a 40 μm filter (Thermo Fisher Scientific, Waltham, MA), and the remaining cells were pre-plated multiple times to eliminate fibroblast contamination. Immediately after purification, myocytes were plated on substrates (prepared as detailed above) at a density of 10^6^ or 10^5^ cells per well in a standard six-well plate for confluent or sparse cultures, respectively. These were incubated at 37°C with a 5% CO_2_ atmosphere. Seeded cultures underwent a wash with PBS 24 hours after plating to remove unattached and dead myocytes. They were then cultured in 10% serum media for another 24 hours at which point the media was changed to 2% serum media. After a total of 72 hours in culture, the samples were fixed and immunostained.

**Fixing, immunostaining and imaging**. After 3–4 days in culture, confluent monolayers of cardiomyocytes were fixed with 4% paraformaldehyde (PFA) (VWR, Radnor, PA) with 0.01% Triton X-100 (Sigma-Aldrich, Inc., Saint Louis, MO) for 10 min, and rinsed three times with PBS in 5-min intervals. Cardiomyocytes were stained with nuclei acid-sensitive dye 4’, 6’-diaminodino-2-phenylinodole (DAPI) (Life Technologies, Carlsbad, CA) for chromatin, FITC-phalloidin (Alexa Fluor 488 Phalloidin, Life Technologies, Carlsbad, CA) for actin, monoclonal mouse sarcomeric anti-*α*-actinin (Sigma-Aldrich, St. Louis, MO), and polyclonal rabbit anti-human fibronectin (Sigma-Aldrich, St. Louis, MO) and incubated for a total of 1–2 hours at room temperature. Secondary staining was applied using tetramethylrhodamine-conjugated goat anti-mouse IgG antibodies (Alexa Fluor 633 Goat anti-mouse, Life Technologies, Carlsbad, CA) and goat anti-rabbit IgG antibodies (Alexa Fluor 750 goat anti-rabbit, Life Technologies, Carlsbad, CA) for a 1–2 hour incubation. After each incubation period, coverslips were rinsed three times with PBS for 5–10 min. Each coverslip was then mounted onto a microscope slide preserved with prolong gold antifade reagent (Life Technologies, Carlsbad, CA). The images were collected using an IX-83 inverted motorized microscope (Olympus America, Center Valley, PA) with an UPLFLN 40x oil immersion objective (Olympus America, Center Valley, PA) and a digital CCD camera ORCA-R2 C10600-10B (Hamamatsu Photonics, Shizuoka Prefecture, Japan). For isotropic monolayers, at least ten fields of view were collected for every sample.

**‘Noiseless” image generation**. A macro was created in ImageJ that allows the user to select regions without imperfections in an image. The regions that were not selected became masked, resulting in a series of images with only regions of interest displayed in the new masked images for every channel imaged (for example: DAPI, m-cherry, GFP)(Fig. 4). The masked images could then be analyzed using the same codes used for raw images.

**Calculating construct angles**. To determine construct angles, we adapted a previous MATLAB code that detects ridges of a fingerprint [20, 25, 26]. This code was used to detect Z-lines and actin fibers in the images. In the code, a binary mask applied to the image determined the constructs and a filter was applied to clean up the constructs that were identified in the images. The code took pixel information from the images and for every non-empty pixel in the image, a pseudo-vector was calculated and used to determine the OOP for Z-lines and actin fibrils, as well as a new set of pseudo-vectors for each square in the grid (Fig. 4B, D) These new pseudo-vectors were then utilized to calculate the COOP between two constructs (i.e. Z-lines and actin fibrils) or two cells (Fig. 4).

**Statistics**. To calculate the average angle between the constructs (⟨*θ*_0_⟩) and the standard deviation of those angles (*σ*_*θ*_0__) across multiple conver-slips, it is essential to keep in mind that the angle period is *π*. The simplest way, but not the only way, to generate ⟨*θ*_0_⟩ and *σ*_*θ*_0__ is to calculate the director of the director pseudo-vectors ${\hat{n}}_{\text{ALL}}$ of each cover-slip. Meaning that in Equation (1) $\vec{k_{i}}={\hat{n}}_{i}$ where *i* is the cover-slip, and the ${\hat{n}}_{\text{ALL}}$ is the the eigenvector of the tensor from Equation (1) that corresponds to the eigenvalue from Equation (2). The angle for each cover-slip is then determined as follows

$$\begin{array}{c} \theta_{0,i}=\left\{\begin{array}{cc} arccos({\hat{n}}_{i}) & \text{for}\;\frac{\pi }{4}<arccos\left({\hat{n}}_{ALL}\right)<\frac{3\pi }{4}\\ arcsin({\hat{n}}_{i}) & \text{for}\;arccos\left({\hat{n}}_{ALL}\right)>\frac{3\pi }{4}\;\text{or}\;arccos\left({\hat{n}}_{ALL}\right)<\frac{\pi }{4}. \end{array}\right. \end{array}$$ (44)

The ⟨*θ*_0_⟩ and *σ*_*θ*_0__ are the average and standard deviation of *θ*_0,*i*_ for all cover-silps. Obviously if the COOP →0 then this procedure is useless as the angles will be inconsistent between the constructs. However, this procedure is a convenient way to determine the range in which it is most convenient to report the angle (i.e. 0→*π* or $-\frac{\pi }{2}$→$\frac{\pi }{2}$). Additionally, there are two possible angles that can be calculated (Fig. 1B). We chose to provide the clock-wise angle from $\vec{p}$ to $\vec{q}$, but it is also possible to calculate only the acute angle instead. To compare the COOP, COOP_c_, and COOP_u_ in the analysis of the experimental data, the one way ANOVA with the Student-Newman-Keuls test was used.

## Supporting Information

S1 Supplemental Text The supporting text provides the reader with more information about standard methods, such as the OOP, circular statistics, and circular correlation. This information is provided in the same notation used for the manuscript.
(PDF)

S1 Fig Example of OOP using synthetic data. For (A-D) schematic of the construct is on the left, and the orientation distribution with the OOP and standard deviation is on the right. (A) Perfect alignment; (B) almost perfect organization; (C) somewhat anisotropic; (D) perfectly isotropic.
(TIF)

S2 Fig Minimum Sample Size. Statistical significance at p<0.05, with OOP error of *σ*_*OOP*_ = 0.1, *σ*_*OOP*_ = 0.3, and *σ*_*OOP*_ = 0.5.
(TIF)

S3 Fig Schematic for grid size selection. For (A-D) schematic of square grids (dashed black line outlines) on Z-lines(red). (A-B) Grid size equivalent to one sarcomere complex length. (C-D) Grid size equivalent to two sarcomere complex length. (A, C) Grid by chance aligns with Z-lines. (B, D) Grid does not align with Z-lines. we choose the grids shown in C-D because we cannot control the alignment of grid to sarcomere complex.
(TIF)

S4 Fig Implementation Flow Chart. Flow chart sketching the implementation of the new method for experimental data. The additional steps for generating “noiseless” images with minimal imperfections is highlighted in blue.
(TIF)

S5 Fig Fibronectin stains. Image of the fibronectin island for each cell in Fig. 4I were cropped such that the triangular islands aligned with each other.
(TIF)

S1 Table Variables and parameters. A table of definitions for all variables and parameters.
(PDF)

S1 Code Code for synthetic data for four limiting cases. Code that creates synthetic data for four limiting cases.
(M) Click here for additional data file.

S2 Code Code for boundaries of normal region of the COOP. Code that creates synthetic data for boundaries of normal region of the COOP.
(M) Click here for additional data file.
