# Supplementary material for: Metrics for Assessing Cytoskeletal Orientational Correlations and Consistency
Source: PLoS Comput Biol. 2015 Apr 7;11(4):e1004190. doi: 10.1371/journal.pcbi.1004190 (PMC4388480; doi:10.1371/journal.pcbi.1004190)
Supplement: S1 Supplemental Text — This information is provided in the same notation used for the manuscript. (PDF) [file pcbi.1004190.s001.pdf]

Supplemental Text S1

Orientational Order Parameter (OOP)

The OOP characterizes the order of orientation of a single construct. For disordered systems the OOP is zero and for perfectly aligned systems it is one. The OOP is calculated by using a set of vectors, $\overrightarrow{p_i}$, and forming a tensor for each of the vectors. The mean tensor is:

$$\mathbb{T} = \left\langle 2\begin{bmatrix} p_{i,x}p_{i,x} & p_{i,x}p_{i,y} \\ p_{i,x}p_{i,y} & p_{i,y}p_{i,y} \end{bmatrix} - \begin{bmatrix} 1 & 0 \\ 0 & 1 \end{bmatrix} \right\rangle = \{Mean\ tensor\}. \quad (1)$$

The OOP is defined as the maximum eigenvalue of the mean tensor

$$\begin{aligned} OOP &= \max\left[\text{eigenvalue}(\mathbb{T})\right] = \{Orientational\ order\ parameter\} \\ &= \left\langle 2\{\overrightarrow{p_i} \cdot \hat{n_p}\}^2 - 1 \right\rangle = \left\langle \cos\left(2(\alpha - \alpha_0)\right) \right\rangle \end{aligned} \quad (2)$$

where $\hat{n_p}$ and α_0 are the director and mean angle, respectively.

Symmetry of OOP

The OOP also has pseudo-vector symmetry and this can be easily shown.To check for symmetry we need to vary the sign of $\overrightarrow{p_i}$ and $\hat{n_p}$. If we change the sign of $\overrightarrow{p_i}$, $\hat{n_p}$ or both we obtain:

$$\left\langle 2\Big\{-\overrightarrow{p_i} \cdot \hat{n_p}\Big\}^2 - 1 \right\rangle = \left\langle 2\Big\{\overrightarrow{p_i} \cdot \big(-\hat{n_p}\big)\Big\}^2 - 1 \right\rangle = \left\langle 2\Big\{-\overrightarrow{p_i} \cdot \big(-\hat{n_p}\big)\Big\}^2 - 1 \right\rangle = \left\langle 2\{\overrightarrow{p_i} \cdot \hat{n_p}\Big\}^2 - 1 \right\rangle. \quad (3)$$

Thus, we will produce the same OOP no matter the sign of $\overrightarrow{p_i}$ and $\hat{n_p}$ therefore the OOP is symmetric.

Second order correlations

The OOP is not able to characterize second order correlations. To prove this define P as:

$$\overrightarrow{p_i} = \left[\cos(\frac{\pi}{2})), \sin(\frac{\pi}{2}))\right] \text{ and } \overrightarrow{p}_{i+n} = \left[\cos(-\frac{\pi}{2}), \sin(-\frac{\pi}{2})\right] \quad (4)$$

for i = 1, ..., n. Thus, ($\hat{n}_p = 0$ and $\alpha_0 = 0$):

$$OOP_P = \sum_{i=1}^{2n} \cos(2\alpha) = n \cdot \cos\left(2\frac{\pi}{2}\right) + n \cdot \cos\left(2\left(-\frac{\pi}{2}\right)\right) = 0. \tag{5}$$

Thus, OOP=0 even though there is obvious organization in P.

Circular Statistics (assume period of π)

It is possible to show that the R of circular statistics [1] is the same as the OOP. If the data is distributed:

$$\alpha = \frac{2\pi x}{k} \tag{6}$$

where, x is the data in the original scale, k is the total number of steps on the x scale, and α is the variable on the new directional scale (i.e. with a standard 2π period). In our case a rod that is β degrees away from the director is physically the same rod as the one $\beta + \pi$ degrees away. Therefore in our case $k = \pi$ and:

$$\alpha = 2\theta \tag{7}$$

where, θ is defined as the angle that we measured from the director. From this it follows:

$$S = \frac{1}{N}\sum_{i=1}^{N} \sin 2\theta_i \tag{8}$$

$$C = \frac{1}{N}\sum_{i=1}^{N} \cos 2\theta_i \tag{9}$$

$$R = \sqrt{S^2 + C^2}. \tag{10}$$

If we assume that the director is orientated such that $\theta_{\hat{n}} = 0$, then the angles are evenly distributed between positive and negative and therefore $S = 0$. We can then write R as:

$$R = C = \frac{1}{N}\sum_{i=1}^{N} \cos 2\theta_i = \langle \cos 2\theta_i \rangle. \tag{11}$$

Therefore, by definition of the director we will have the range $0 < R < 1$ and it is equivalent to the $OOP = 2\langle \cos^2 \theta_i \rangle - 1 = \langle \cos 2\theta_i \rangle = R$.

Circular Correlation

In the special case, with both constructs having a uniform distribution, i.e. both being perfectly isotropic, the correlation coefficient and COOP converge to the same equation. If the angles are uniformally distributed on the circle the correlation coefficient [2] can be written as

$$r = \sqrt{\left(\frac{1}{N}\sum_{i=1}^{N}\cos 2\theta_i\right)^2 + \left(\frac{1}{N}\sum_{i=1}^{N}\sin 2\theta_i\right)^2} \tag{12}$$

where θ represents the angle between two biological constructs. If the director is to be assumed $\hat{n} = [1, 0]$ then $\langle \sin 2\theta_i \rangle = 0$, and therefore

$$r = \sqrt{\left(\frac{1}{N}\sum_{i=1}^{N}\cos 2\theta_i\right)^2} = \langle \cos 2\theta_i \rangle. \tag{13}$$

Thus, $COOP = 2\langle \cos^2 \theta_i \rangle - 1 = \langle \cos 2\theta_i \rangle = r$.

References

1. Berens P (2009) Circstat: A matlab toolbox for circular statistics. J Stat Softw 31: 1-21.

2. Batschelet E (1981) Circular Statistics in Biology. London: Academic Press, 371 pp.
