# Supplementary figures and images for: Metrics for Assessing Cytoskeletal Orientational Correlations and Consistency
Source: PLoS Comput Biol. 2015 Apr 7;11(4):e1004190. doi: 10.1371/journal.pcbi.1004190 (PMC4388480; doi:10.1371/journal.pcbi.1004190)

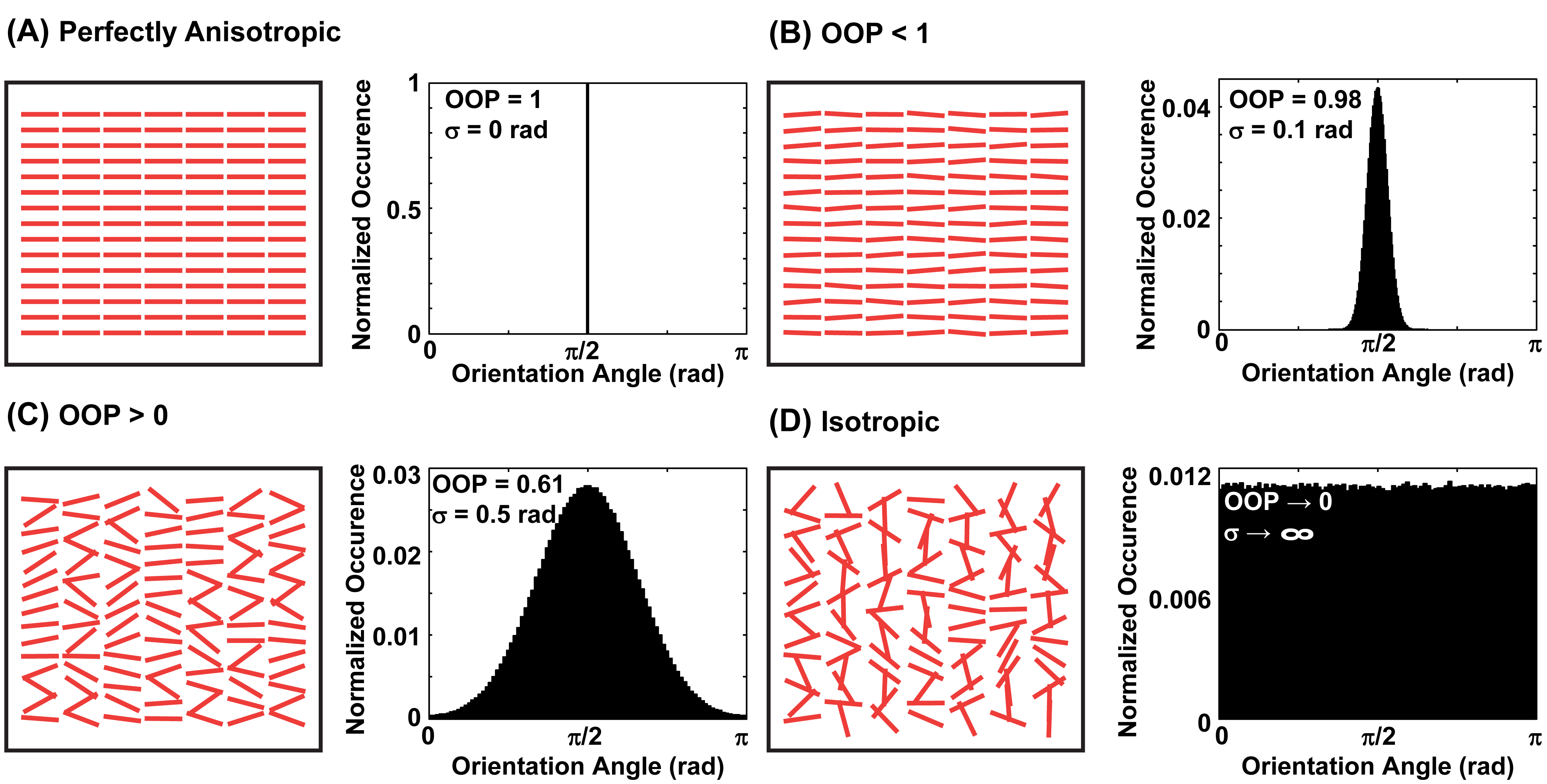

Supplement: S1 Fig — For (A-D) schematic of the construct is on the left, and the orientation distribution with the OOP and standard deviation is on the right. (A) Perfect alignment; (B) almost perfect organization; (C) somewhat anisotropic; (D) perfectly isotropic. (TIF) [file pcbi.1004190.s002.tif]

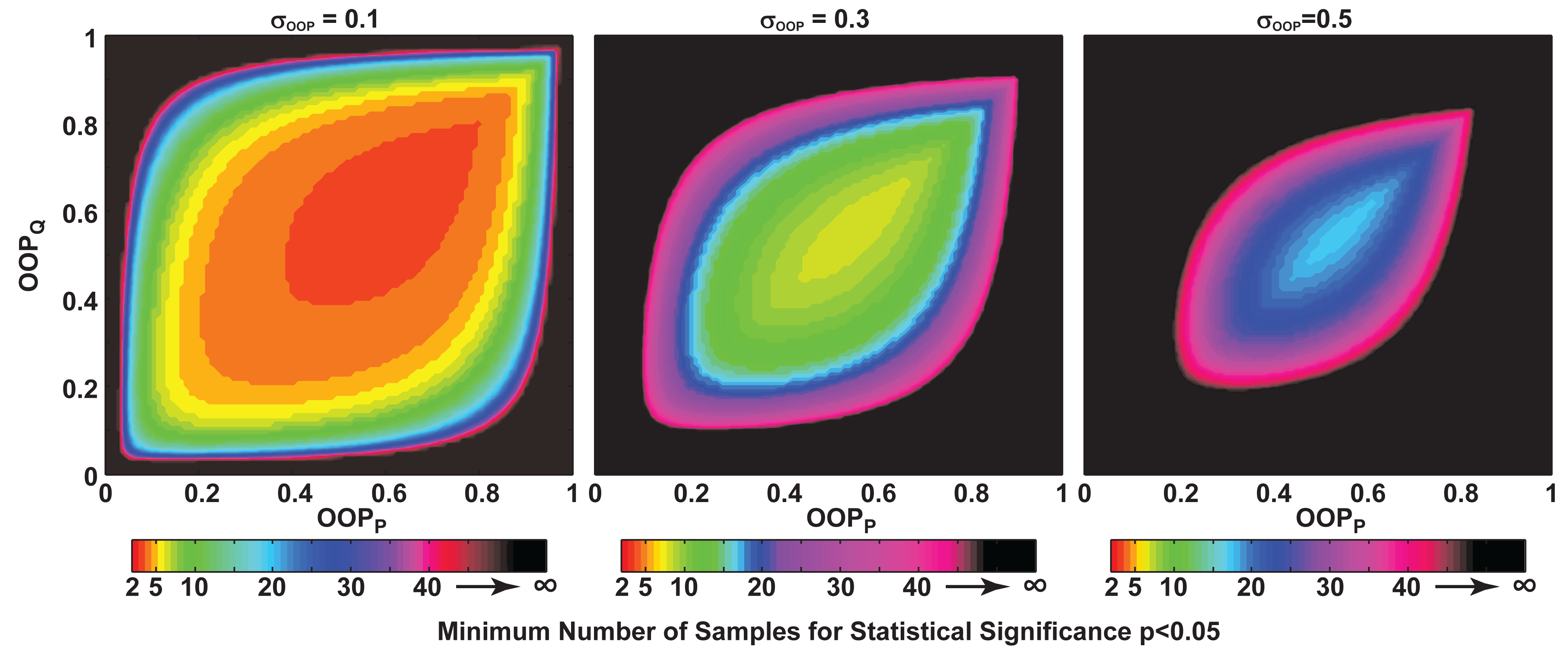

Supplement: S2 Fig — Statistical significance at p<0.05, with OOP error of σ OOP = 0.1, σ OOP = 0.3, and σ OOP = 0.5. (TIF) [file pcbi.1004190.s003.tif]

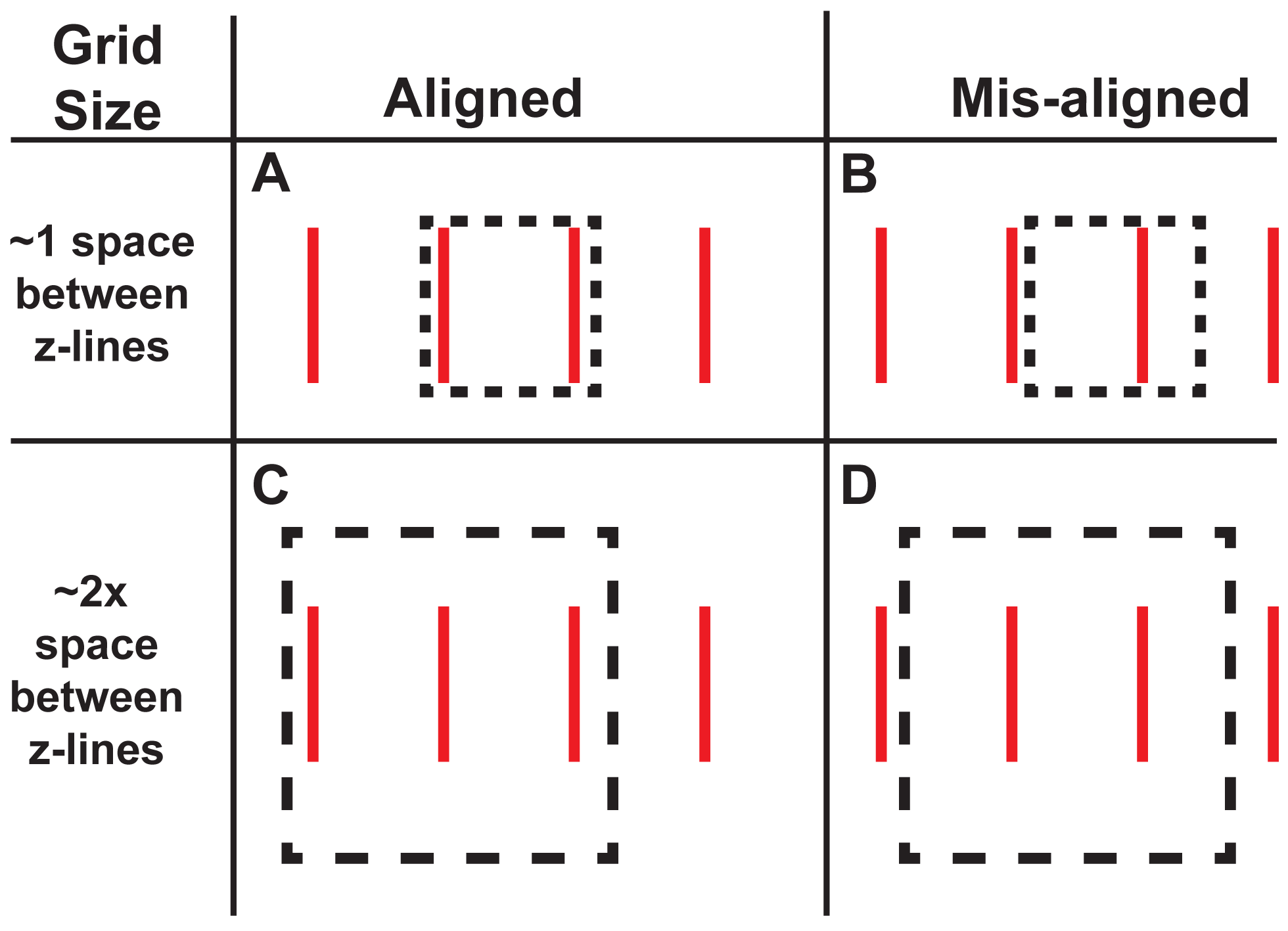

Supplement: S3 Fig — For (A-D) schematic of square grids (dashed black line outlines) on Z-lines(red). (A-B) Grid size equivalent to one sarcomere complex length. (C-D) Grid size equivalent to two sarcomere complex length. (A, C) Grid by chance aligns with Z-lines. (B, D) Grid does not align with Z-lines. we choose the grids shown in C-D because we cannot control the alignment of grid to sarcomere complex. (TIF) [file pcbi.1004190.s004.tif]

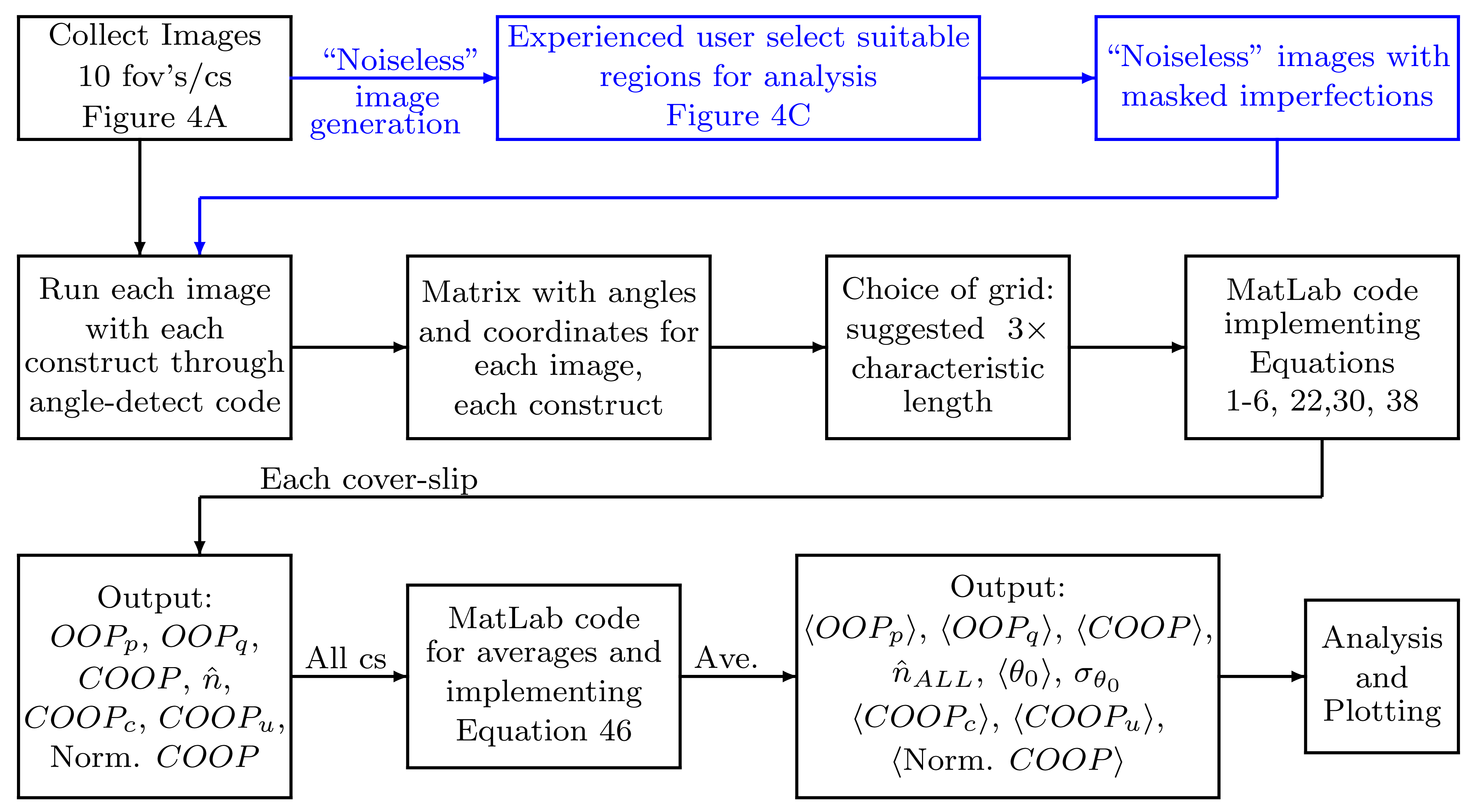

Supplement: S4 Fig — Flow chart sketching the implementation of the new method for experimental data. The additional steps for generating “noiseless” images with minimal imperfections is highlighted in blue. (TIF) [file pcbi.1004190.s005.tif]

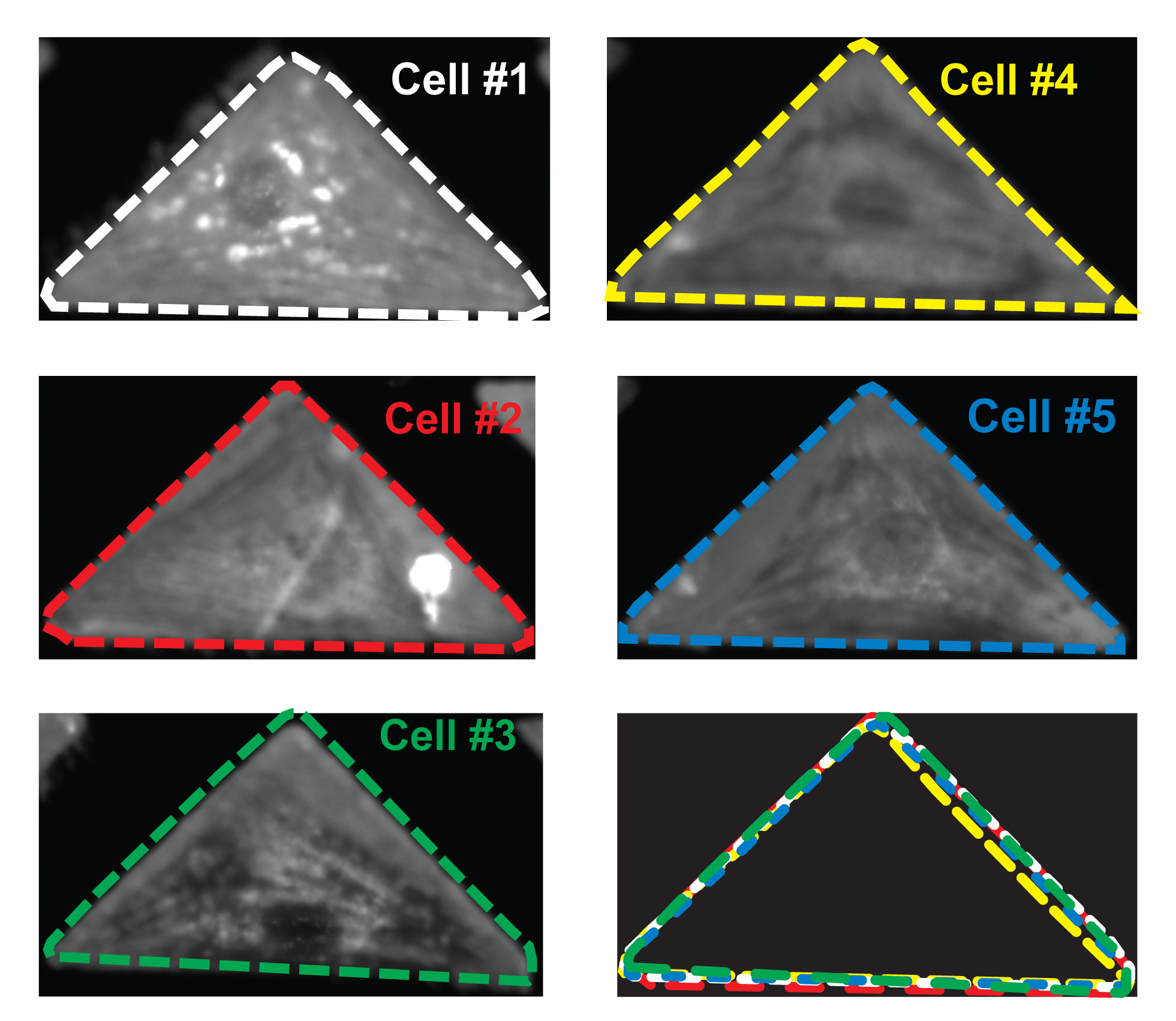

Supplement: S5 Fig — Image of the fibronectin island for each cell in Fig. 4I were cropped such that the triangular islands aligned with each other. (TIF) [file pcbi.1004190.s006.tif]
