# Supplementary material for: Metrics for Assessing Cytoskeletal Orientational Correlations and Consistency
Source: PLoS Comput Biol. 2015 Apr 7;11(4):e1004190. doi: 10.1371/journal.pcbi.1004190 (PMC4388480; doi:10.1371/journal.pcbi.1004190)
Supplement: S1 Table — A table of definitions for all variables and parameters. (PDF) [file pcbi.1004190.s007.pdf]

Table S1. Variables and parameters

parameters and variables	definition
COOP	co-orientational order parameter
OOP	orientational order parameter
P	construct P, the first construct
$\overrightarrow{q_i}$	a set of pseudo vectors for construct P
$p_{i,x}$	a set of pseudo vectors for construct P for the x component
$p_{i,y}$	a set of pseudo vectors for construct P for the y component
Q	construct Q, the second construct
$\overrightarrow{q_i}$	a set of pseudo vectors for construct Q
$q_{i,x}$	a set of pseudo vectors for construct Q for x
$q_{i,y}$	a set of pseudo vectors for construct Q for y
$\mathbb{T}_K$	mean order tensor
OOP_K	orientational order construct of K
K	either construct P or construct Q
$\overrightarrow{k_i}$	either a set of pseudo vectors for either construct P or construct Q
$k_{i,x}$	the x component of the pseudo vector
$k_{i,y}$	the y component of the pseudo vector
F	new field that represents the angle (θ) between the two constructs P and Q
θ	angle between $\overrightarrow{p_i}$ and $\overrightarrow{q_i}$ or random noise
$\overrightarrow{f_i}$	a set of pseudo vectors for the COOP
$f_{i,x}$	the x position of the pseudo vector
$f_{i,y}$	the y position of the pseudo vector
$\mathbb{T}_{PQ}$	mean order tensor of the system
$COOP_{PQ}$	co-orientational order parameter of P and Q
$\hat{n}$	the director
$f'_{i,x}$	the x position of the pseudo vector with the switch of P and Q
$f'_{i,x}$	the y position of the pseudo vector with the switch of P and Q
$COOP_{QP}$	co-orientational order parameter of Q and P
ν	angle used to rotated field
Q_{rot}	rotated construct Q
$\overrightarrow{q}_{i,rot}$	a rotated set of pseudo vectors for construct Q_{rot}
F_{rot}	rotated field F
$\overrightarrow{f}_{i,rot}$	rotated vectors $\overrightarrow{f_i}$
$\langle f_{i,rot,x} \rangle$	the mean of the rotated vectors in the x position
$\langle f_{i,rot,y} \rangle$	the mean of the rotated vectors in the y position
$COOProt$	the rotated COOP
$\hat{n}_p$	the director of construct P
$\hat{n}_q$	the director of construct Q
θ_0	the mean angle between P and Q
α	angle of the vector $\overrightarrow{p}$
β	angle of the vector $\overrightarrow{q}$
$COOP_u$	uncorrelated COOP
COOP anti-cor	anti-correlated COOP
n	$\{1, 2, \dots\}$
$COOP_c$	correlated COOP
COOP ultra-cor	ultra-correlated COOP
α_i	angles of vectors
θ_i	angles of vectors
i	$\{1, \dots, n\}$
Normalized COOP	the normalized COOP value
σ_{COOP_u}	standard deviation of the uncorrelated COOP
σ_{COOP_c}	standard deviation of the correlated COOP
σ_{OOP}	the OOP error
N	sample size
p	p-value
W_i	weight
ρ	density
$COOP_{sarc}$	the COOP of Z-lines for each cell wise comparison
$COOP_{actin}$	the COOP of actin for each cell wise comparison
